# Stage at Diagnosis and International Survival Variation in Childhood Tumors in the BENCHISTA Study

**DOI:** 10.1001/jamanetworkopen.2025.56747

**Published:** 2026-02-09

**Authors:** Laura Botta, Fabio Didonè, Angela Lopez-Cortes, Adela Cañete Nieto, Emmanuel Desandes, Lisa L. Hjalgrim, Zsuzsanna Jakab, Charles A. Stiller, Bernward Zeller, Simon Bailey, Nathalie Gaspar, Filippo Spreafico, Sandra J. Strauss, Gemma Gatta, Kathy Pritchard-Jones

**Affiliations:** 1 Evaluative Epidemiology Unit, Department of Epidemiology and Data Science, Fondazione IRCCS Istituto Nazionale dei Tumori di Milano, Milan, Italy; 2 UCL Developmental Biology & Cancer Research Department, UCL GOS Institute of Child Health, London, United Kingdom; 3 Paediatric Oncology and Hematology Unit, Hospital UiP La Fe, Department of Pediatrics, Obstetrics and Gynecology, University of Valencia, Spain; 4 National Registry of Childhood Cancers, CRESS, UMRS 1153, INSERM, Paris-Cité University, France; 5 National Registry of Childhood Solid Tumors, Institut de Cancérologie de Lorraine, Vandœuvre-lès-Nancy, France; 6 Department of Paediatrics and Adolescent Medicine, University of Copenhagen, Rigshospitalet, Copenhagen, Denmark; 7 National Childhood Cancer Registry (NCCR), Hungarian Pediatric Oncology Network (HuPON), Budapest, Hungary; 8 Department of Pediatrics, Semmelweis University, Budapest, Hungary; 9 National Disease Registration Service, Transformation Directorate, NHS England, London, United Kingdom; 10 Department of Pediatric Hematology-Oncology, Oslo University Hospital, Oslo, Norway; 11 Sir James Spence Institute, Royal Victoria Infirmary, Newcastle upon Tyne, United Kingdom; 12 Département de cancérologie de l’enfant et l’adolescent, Gustave Roussy, Villejuif, France; 13 Oncology Unit, IRCCS Istituto Giannina Gaslini, Genoa, Italy; 14 S.C. Pediatria Oncologica, Fondazione IRCCS Istituto Nazionale dei Tumori, Milan, Italy; 15 UCL Cancer Institute, London, United Kingdom

## Abstract

**Importance:**

Understanding the reasons for variations in population-level survival differences in childhood cancer is important to guide improvement efforts. Collaboration between population-based cancer registries (CRs) to apply the international consensus Toronto guidelines to record tumor stage at diagnosis is a key first step.

**Objective:**

To test whether survival probabilities by tumor stage vary internationally, using 6 childhood solid tumors as exemplars.

**Design, Setting, and Participants:**

The International Benchmarking of Childhood Cancer Survival by Stage (BENCHISTA) population-based retrospective cohort study included all incident cases of neuroblastoma, Wilms tumor, medulloblastoma, osteosarcoma, Ewing sarcoma of bone, and rhabdomyosarcoma diagnosed between January 1, 2014, and December 31, 2017, with 3-year follow-up for survival. A total of 73 CRs from 27 countries (23 European as well as Australia, Brazil, Canada, and Japan) constituted the dataset. Analyses were conducted from June 2023 to December 2024.

**Main Outcomes and Measures:**

Three-year overall survival (OS) by stage for each tumor type, with comparisons between countries grouped into 5 predefined European areas. Multivariable Cox and logistic models estimated each area’s hazard or odds ratio of death compared with Central Europe (Austria, Belgium, France, Germany, Switzerland, and the Netherlands), adjusted by age group and stage.

**Results:**

A total of 9883 cases were included; 4452 (45%) were girls and overall median (IQR) age was 54 (22-122) months; stage completeness was 93% (9199 of 9883). Three-year OS rates were as follows: Wilms tumor, 95% (95% CI, 94%-96%); neuroblastoma, 83% (95% CI, 81%-84%); medulloblastoma, 79% (95% CI, 77%-81%); Ewing sarcoma, 78% (95% CI, 75%-80%); rhabdomyosarcoma, 77% (95% CI, 74%-79%); and osteosarcoma, 75% (95% CI, 73%-77%). Geographical variations in age-adjusted OS were found for neuroblastoma, medulloblastoma, Ewing sarcoma, and rhabdomyosarcoma. Following additional adjustment for stage, differences were no longer significant for neuroblastoma (in the UK and Ireland) and rhabdomyosarcoma (in Eastern Europe) while becoming significant for neuroblastoma in Eastern Europe (hazard ratio, 1.36; 95% CI, 1.05-1.76) and medulloblastoma in Southern Europe (hazard ratio, 1.42; 95% CI, 1.03-1.94). However, no mitigation of survival variation was observed for Ewing sarcoma in the UK and Ireland (hazard ratio, 2.06; 95% CI, 1.39-3.04) and Eastern Europe (hazard ratio, 1.87; 95% CI, 1.22-2.86) as well as for medulloblastoma in Eastern Europe (hazard ratio, 1.68; 95% CI, 1.13-2.49).

**Conclusions and Relevance:**

In this BENCHISTA cohort study of 6 solid tumors, international variation in population-level OS was associated with differences in tumor stage distribution for some cancer types and regions. Additional factors are suggested for further investigation. The results have important implications for national health systems for monitoring early diagnosis efforts and supporting collaboration between CRs and clinicians to sustain standardized use of Toronto guidelines to improve understanding of survival variation in childhood cancer.

## Key Points

**Question:**

Are differences in tumor stage at diagnosis associated with population-level survival disparities for 6 childhood solid tumors across regions?

**Findings:**

This cohort study with 9883 cases from 73 cancer registries in 27 countries found that 3-year overall survival decreased with higher tumor stage at diagnosis. Regional survival differences were partly associated with stage variation, notably in neuroblastoma, rhabdomyosarcoma, and medulloblastoma, but not Ewing sarcoma.

**Meaning:**

The study highlights geographic disparities in survival, stressing the need for early diagnosis, precise staging, and stronger collaboration between clinicians and cancer registries to understand survival gaps.

## Teaser

This cohort study reports the findings of the BENCHISTA project, which evaluated whether stage at diagnosis is associated with different rates of survival among children with cancer.

## Introduction

Survival from childhood cancer is known to vary internationally, with significant differences between the least- and best-resourced countries.^1,2^ Even within Europe, where most countries are classified as high income, geographical variation in survival is reported; however, population-based data indicate that these differences are gradually reducing over time.^3,4,5^ These studies, which highlight differences in overall survival between geographical areas, emphasize the importance of population-based data from cancer registries (CRs) in enabling pediatric oncologists and health policymakers to investigate possible reasons for this variation. Specifically, for Europe, 5-year overall survival was lower in Eastern countries and higher in Northern and some Central European countries.^3,4,5^ Possible reasons for this include variation in stage at diagnosis; quality of diagnosis and risk stratification; nonstage prognostic factors (NSPs), including tumor biology and genetics; treatments given; and supportive care for treatment toxic effects.^6,7^ Of these, tumor stage at diagnosis is considered a key prognostic factor across most cancers in children and adults.^8,9^

Unfortunately, information on stage at diagnosis collected by population-based CRs for childhood cancers is often missing or incomplete and not collected in a standardized way; this is because staging systems developed for adult cancers are generally not applicable to pediatric tumors. Population-level survival by stage for childhood solid tumors has been reported recently but only at a national or regional level.^8,10,11,12^ The development of the internationally recognized Toronto Stage Guidelines (TG)^6,7,12,13,14^ in 2014 provided an impetus and opportunity to collect stage at a population level in a format suitable for reliable international survival comparisons.

The International Benchmarking of Childhood Cancer Survival by Stage (BENCHISTA) Project aims to better understand the reasons underlying variation in childhood cancer survival between countries and to identify areas for improvement. By focusing on stage at diagnosis—crucial for determining treatment intensity and prognosis—the project encourages the use of TG for standardized recording stage at diagnosis for childhood cancers by CRs. This collaborative effort selected 6 pediatric solid tumors for which overall survival has been shown to vary between geographies, including within some multinational clinical trials.^3,15,16,17,18,19,20^

Furthermore, these cancers have unambiguous coding for identification by CRs and are included in the TG. The research questions of the project were 2-fold: first, to determine whether childhood cancers are more likely to be diagnosed at a more advanced stage in some areas compared with others and, second, whether by adjusting for stage, any observed disparities in survival would be mitigated. The first research question was recently reported by Botta et al,^21^ demonstrating that the distribution of stage at diagnosis assessed at a population level shows significant variation between geographical areas for 4 of these 6 childhood tumors across European geographical areas and for some non-European countries. This article presents analysis of the association of stage variation with geographical differences in survival and survival by stage, using population-based CRs and 6 selected childhood solid tumors.

## Methods

### Study Design and Data Collection

Seventy-three CRs from 23 European countries, Australia, Brazil, Canada, and Japan participated in this retrospective population-based cohort study (eTable 1 in Supplement 1). A total of 10 366 cases of the 6 specified childhood solid tumors (age 0-14 years: neuroblastoma, Wilms tumor, and medulloblastoma; age 0-17 years: osteosarcoma, Ewing sarcoma, and rhabdomyosarcoma) diagnosed during at least 3 consecutive years between January 1, 2014, and December 31, 2017, and followed up for 3 years after diagnosis, were submitted.^22^

Participating countries had national population coverage except for Italy, Australia, Brazil, Canada and Japan, who submitted only regional data, and Poland, where data were provided by a clinical registry that identifies cases through the national network of designated centers (eTable 1 in Supplement 1). Stage at diagnosis was defined according to TG tier 2 for all tumors except for neuroblastoma, which was analyzed using tier 1, as the German Childhood CR could not provide tier 2 stages because image-defined risk factors were not accessible to this CR.^21^ Due to a lack of access to necessary clinical records from tumor-specific clinical registries, Germany was also unable to provide data on Ewing sarcoma and rhabdomyosarcoma cases. To ensure comparability of tumor stage, the BENCHISTA project supported standardized stage assignment through online training workshops led by clinical experts, data quality assurance audits, and a helpdesk with questions and answers published on the project’s website.^23,24^

The project involved parents of children with cancer, who are represented in the Project Working Group and the Independent Advisory Board. Participating CRs are on the project’s website.^24^

Ethical approval was provided by the institutional review boards of the joint data controllers (University College London and the Fondazione IRCCS Istituto Nazionale dei Tumori di Milano) and local ethics committees where required. Data access, collation, and transfer complied with country-specific laws and regulations. The collection and use of population-based CR data does not require informed consent in most countries. This study followed the Strengthening the Reporting of Observational Studies in Epidemiology (STROBE) reporting guideline for cohort studies.

### Statistical Analysis

The Kaplan-Meier estimator was used to provide nonparametric survival estimates along with their 95% CIs by geographical area and stage, allowing visual comparison through survival curves. The overall BENCHISTA pool was used for overall survival curves by stage, excluding low-quality follow-up data (Table and eTable 1 in Supplement 1). European countries were grouped as in previous EUROCARE^5^ studies and as applied in BENCHISTA^21^ (eTable 1 in Supplement 1), while non-European countries were considered individually. Central Europe included Austria, Belgium, France, Germany, the Netherlands, and Switzerland; Eastern Europe: Bulgaria, Czechia, Estonia, Hungary, Poland, and Romania; Northern Europe: Denmark, Norway, and Sweden; Southern Europe: Greece, Italy, Malta, Portugal, Slovenia, and Spain; and the UK and Ireland: Northern Ireland, Ireland, England, Scotland, and Wales. Overall survival rates for BENCHISTA Europe includes only European data.

**Table.** Number of Cases, Stage Completeness, and Data Quality Indicators by Tumor Type and Geographical Area

Area or country	Total cases, No.		Excluded cases, No. (%)^a^	Included cases, No. (%)	Data quality indicators, No. (%)	
					Stage completeness	Lost to follow-up^b^
**All tumor types**						
Overall	9941	58 (1)		9883 (99)	9199 (93)	149 (2)
**Neuroblastoma, age 0-14 y**						
Australia	76	0		76 (100)	62 (82)	0
Brazil^c^	65	2 (3)		63 (99)	61 (97)	9 (24)^d^
Canada	115	0		115 (100)	115 (100)	1 (1)
Japan^e^	68	2 (3)		66 (97)	60 (91)	8 (13)^d^
Central Europe	1083	0		1083 (100)	999 (92)	19 (2)
Eastern Europe	491	2 (<1)		489 (>99)	487 (100)	15 (4)
Northern Europe	102	0		102 (100)	102 (100)	0
Southern Europe	589	4 (1)		585 (99)	572 (98)	5 (1)
United Kingdom and Ireland	416	1 (<1)		415 (>99)	386 (93)	0
Total	3005	11 (<1)		2994	2844 (95)	57 (2)
**Wilms tumor, age 0-14 y**						
Australia	66	0		66 (100)	63 (95)	0
Brazil^c^	126	7 (6)		119 (94)	106 (89)	23 (24)^d^
Canada	63	0		63 (100)	63 (100)	1 (2)
Japan^e^	26	2 (8)		24 (92)	24 (100)	0
Central Europe	797	0		797 (100)	736 (92)	6 (1)
Eastern Europe^f^	156	0		156 (100)	154 (99)	1 (1)
Northern Europe	102	0		102 (100)	102 (100)	1 (1)
Southern Europe	343	5 (1)		338 (99)	311 (92)	1 (<1)
United Kingdom and Ireland	425	1 (<1)		424 (>99)	403 (95)	1 (<1)
Total	2104	15 (1)		2089 (99)	1962 (94)	34 (2)
**Medulloblastoma, age 0-14 y**						
Australia	44	0		44 (100)	36 (82)	0
Brazil^c^	69	4 (6)		65 (94)	60 (92)	7 (16)^d^
Canada	50	0		50 (100)	50 (100)	0
Japan	19	0		19 (100)	18 (95)	2 (11)
Central Europe	578	0		578 (100)	535 (93)	9 (2)
Eastern Europe^f^	120	0		120 (100)	115 (96)	0
Northern Europe	62	0		62 (100)	61 (98)	0
Southern Europe	254	3 (1)		251 (99)	221 (88)	4 (2)
United Kingdom and Ireland	238	1 (<1)		237 (>99)	183 (77)	0
Total	1434	8 (1)		1426 (99)	1279 (90)	22 (2)
**Osteosarcoma, age <18 y^g^**						
Australia	NE	NE		NE	NE	NE
Brazil^a^	90	8 (9)		82 (91)	74 (90)	7 (17)^d^
Canada	48	0		48 (100)	47 (98)	2 (6)
Japan	12	0		12 (100)	11 (92)	0
Central Europe	572	0		572 (100)	491 (86)	3 (1)
Eastern Europe^c^	98	0		98 (100)	98 (100)	0
Northern Europe	43	0		43 (100)	43 (100)	0
Southern Europe	221	0		221 (100)	215 (97)	1 (1)
United Kingdom and Ireland	230	1 (<1)		229 (99)	209 (91)	0
Total	1314	9 (1)		1305 (99)	1188 (91)	13 (1)
**Ewing sarcoma, age <18 y^g^**						
Australia	NE	NE		NE	NE	NE
Brazil^c^	41	1 (2)		40 (98)	37 (93)	8 (35)^d^
Canada	24	0		24 (100)	23 (96)	0
Japan	5	0		5 (100)	5 (100)	0
Central Europe	307	0		307 (100)	304 (99)	0
Eastern Europe^f^	107	0		107 (100)	107 (100)	0
Northern Europe	43	0		43 (100)	43 (100)	0
Southern Europe	208	1 (<1)		207 (>99)	196 (95)	0
United Kingdom and Ireland	162	1 (1)		161 (99)	149 (93)	0
Total	897	3 (<1)		894 (>99)	864 (97)	8 (1)
**Rhabdomyosarcoma, age <18 y^g^**						
Australia	NE	NE		NE	NE	NE
Brazil^c^	72	7 (10)		65 (90)	56 (86)	9 (30)^d^
Canada	58	0		58 (100)	58 (100)	2 (5)
Japan^e^	21	1 (5)		20 (95)	20 (100)	2 (12)^d^
Central Europe	354	0		354 (100)	328 (93)	0
Eastern Europe^f^	124	0		124 (100)	121 (98)	2 (2)
Northern Europe	57	0		57 (100)	57 (100)	0
Southern Europe	203	1 (<1)		202 (>99)	188 (93)	0
United Kingdom and Ireland	298	3 (1)		295 (99)	234 (79)	0
Total	1187	12 (1)		1175 (99)	1062 (90)	15 (2)

Abbreviation: NE, not eligible.

^a^ Cases were excluded when missing vital status or follow-up duration.

^b^ Patients whose last known vital status was alive and who were censored within 3 years of diagnosis divided by the patients who are alive.

^c^ Brazil results are included only for area-specific descriptive analyses of all 6 cancers (eTable 4 in Supplement 1) and excluded from aggregated and comparative analyses, as it had higher proportions of cases with incomplete follow-up (eTable 1 in Supplement 1).

^d^ Percentages equal to or greater than 12%.

^e^ Excludes Japan from aggregated and comparative survival analysis for neuroblastoma and rhabdomyosarcoma (eTable 4 in Supplement 1), as it had higher proportions of cases with incomplete follow-up (eTable 1 in Supplement 1).

^f^ Excludes Poland from aggregated and comparative survival analysis for all cancer types except neuroblastoma, as the Polish clinical registry had higher proportions of cases (>12%) with incomplete follow-up (eTable 1 in Supplement 1).

^g^ Sarcomas are calculated excluding data from Australia, Greece, Registro Español de Tumores Infantiles (RETI-SEHOP), and Denmark cancer registries, as they did not routinely collect cases among patients older than 15 years (eTable 1 in Supplement 1). Data on Ewing sarcoma and rhabdomyosarcoma cases from Germany were not made available to the project.

To evaluate whether geographical differences in death risks change after adjusting for stage and age group, Cox proportional hazard models were used for all cancers. The proportional hazards assumption was tested with Schoenfeld residuals. When the assumption was met, the hazard ratio (HR) of death was reported; otherwise, to account for nonproportional hazards in age group for neuroblastoma and medulloblastoma we applied stratification by age. This approach allowed the baseline hazard to vary across age groups, without estimating a separate coefficient for age groups and including the other covariates as fixed effects. So, the HRs for medulloblastoma and neuroblastoma were adjusted for age through stratification. For osteosarcoma, where the geographical area is the main exposure of interest and exhibits nonproportional hazards, stratification was not feasible. Therefore, we applied a multivariable logistic model with 3-year survival as the outcome, estimating the odds ratio (OR) of death. For this cancer, the censored cases were less than 1%; nevertheless, to address potential bias due to right censoring, we applied inverse probability of censoring weighting, which weights each observation by the estimated probability of not being censored.^25^ HRs and ORs were calculated using Central Europe as the reference category. Given the well-established prognostic role of age in the literature and confirmed in our data, age was included in all models. In contrast, the prognostic role of sex is less consistent in the literature, and we investigated its role in our data using the likelihood ratio test.

For medulloblastoma, stages M1 to M4 were grouped into a single metastatic category (M+) due to low case numbers. In UK and Ireland medulloblastoma cases, the English CR suspected nonrandom missingness. We confirmed this, as survival among cases with missing cerebrospinal fluid (CSF) results was similar to those classified as stage M0. Sensitivity analyses were performed to assess potential bias from missing stage data and to test the robustness of main findings. No similar concerns were raised for other tumor types and regions, and missingness appeared consistent with a random pattern.

In all countries and cancer types, cases lost to follow-up were included in survival analyses but censored at the date of last contact—except in registries with unreliable follow-up, defined as more than 12% of cases lost or with unknown vital status. These registries were excluded from comparative and aggregated analyses, affecting all cancers in Brazil, neuroblastoma and rhabdomyosarcoma in Japan, and all cancers except neuroblastoma in Poland (eTable 1 in Supplement 1). Stage-specific descriptive survival estimates for Brazil and Japan are still provided in eTable 4 in Supplement 1 to support global representation, highlight disparities, and encourage improvements in cancer data systems, despite potential imprecision related to follow-up limitations.

Stata version 14 (Stata Corp) was used for the analysis. Two-tailed tests were used to assess differences between geographical groups without assuming the direction of the effect. *P* values less than .05 were considered statistically significant.

## Results

A total of 9883 cases, representing 99.4% of the total of 9941 cases from by 27 countries and 73 CRs, were included in the survival analyses (Table). Details of the number of cases by geographical area, stage completeness according to the TG tier used in the analysis, percentage lost to follow-up, and excluded cases due to missing vital status or follow-up are reported in the Table and in the flow chart in eFigure 2 in Supplement 1. In our data, 4452 cases (45%) were among girls, with an overall median age of 54 (22-122) months. Overall stage completeness was 93% (9199 of 9883); 2844 of 3005 (95%) for neuroblastoma, tier 1; and between 90% and 97% for the remaining 5 tier-2 tumors (Table). In European countries, less than 3% of cases were lost to follow-up or had missing vital status. Submitted case counts and data quality indicators by country are detailed in eTable 1 in Supplement 1.

As the non-European countries had either small numbers, incomplete follow-up, or both, we restricted estimation of 3-year overall survival of the 6 tumors to European data in BENCHISTA (ie, excluding Japan, Australia, Brazil, and Canada); this also aids comparison with other European studies. The highest survival was observed for Wilms tumor at 95% (95% CI, 94%-96%) and for neuroblastoma at 83% (95% CI, 81%-84%) (eTable 2 in Supplement 1). Lower survival figures were observed for medulloblastoma, with survival of 79% (95% CI, 77%-81%), and for Ewing sarcoma (78%; 95% CI, 75%-80%), rhabdomyosarcoma (77%; 95% CI, 74%-79%), and osteosarcoma (75%; 95% CI, 73%-77%) (eTable 2 in Supplement 1).

For all 6 tumor types, there was a clear pattern of decreasing survival with increasing tumor stage, confirming stage as a key prognostic factor in population-based data (eFigure 3 and eTable 3 in Supplement 1). The larger gap between 3-year overall survival for patients with stage I and metastatic disease was observed for rhabdomyosarcoma, with survival of 95% (95% CI, 92%-97%) and 45% (95% CI, 39%-50%), respectively. The lowest was found for Wilms tumor, with 99% (95% CI, 98%-99%) for stage I and 87% (95% CI, 83%-90%) for stage IV.

**Figure 1.**
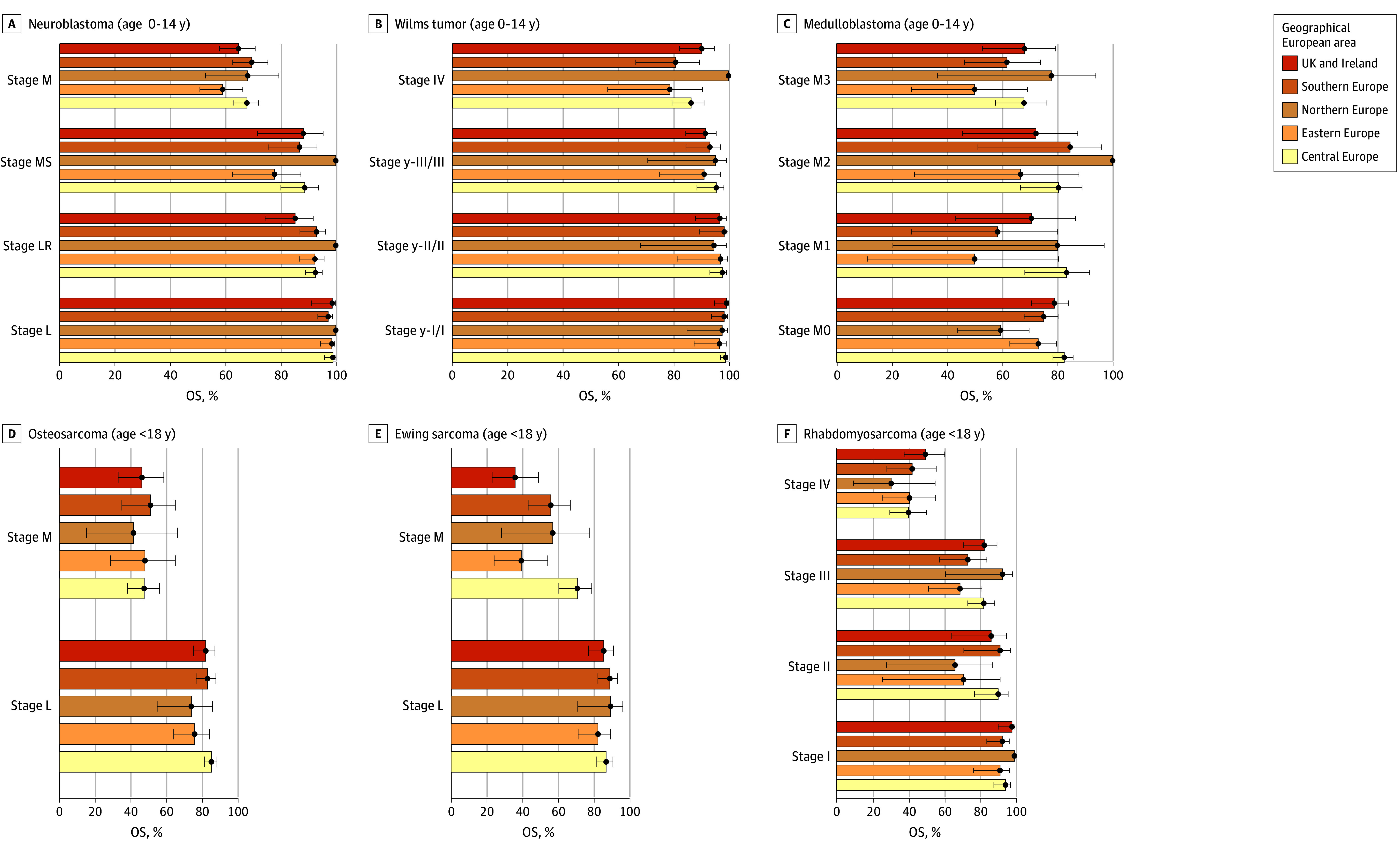
Three-Year Overall Survival by Toronto Stage for Each Tumor Type and European Geographical Area Stage definitions are according to the Toronto guidelines.^6,7^ Neuroblastoma was staged at tier 1; all other tumors were staged at tier 2. For full definitions of stages by tumor type, refer to eFigure 3 in Supplement 1. For neuroblastoma, L indicates localized; LR, locoregional; M, metastatic; MS, metastatic with specific pattern of metastases in children younger than 18 months. For Wilms tumor, y indicates that the tumor was staged after preoperative chemotherapy. For medulloblastoma, M0 indicates cerebrospinal fluid negative for metastases; M1, tumor cells in cerebrospinal fluid; M2, visible metastasis in brain; and M3, visible metastases in spine or in cervicomedullary junction. For osteosarcoma and Ewing sarcoma, L indicates localized; and M, metastatic.

Three-year overall survival and 95% CIs for each tumor type by stage and by geographic area are presented in Figure 1 (European areas only) and eTable 4 in Supplement 1 (all areas). Due to incomplete follow-up in Brazil, only descriptive analyses are presented. For all tumors, geographical variation was less evident for localized (L) stage and most prominent for metastatic (M) stage. Indeed, the only instance of nonoverlapping 95% CIs was for Ewing sarcoma stage M in the UK and Ireland (36%; 95% CI, 23%-49%) and Eastern Europe (39%; 95% CI, 24%-54%) compared with Central Europe (71%; 95% CI, 61%-79%) (Figure 1; eTable 4 in Supplement 1).

**Figure 2.**
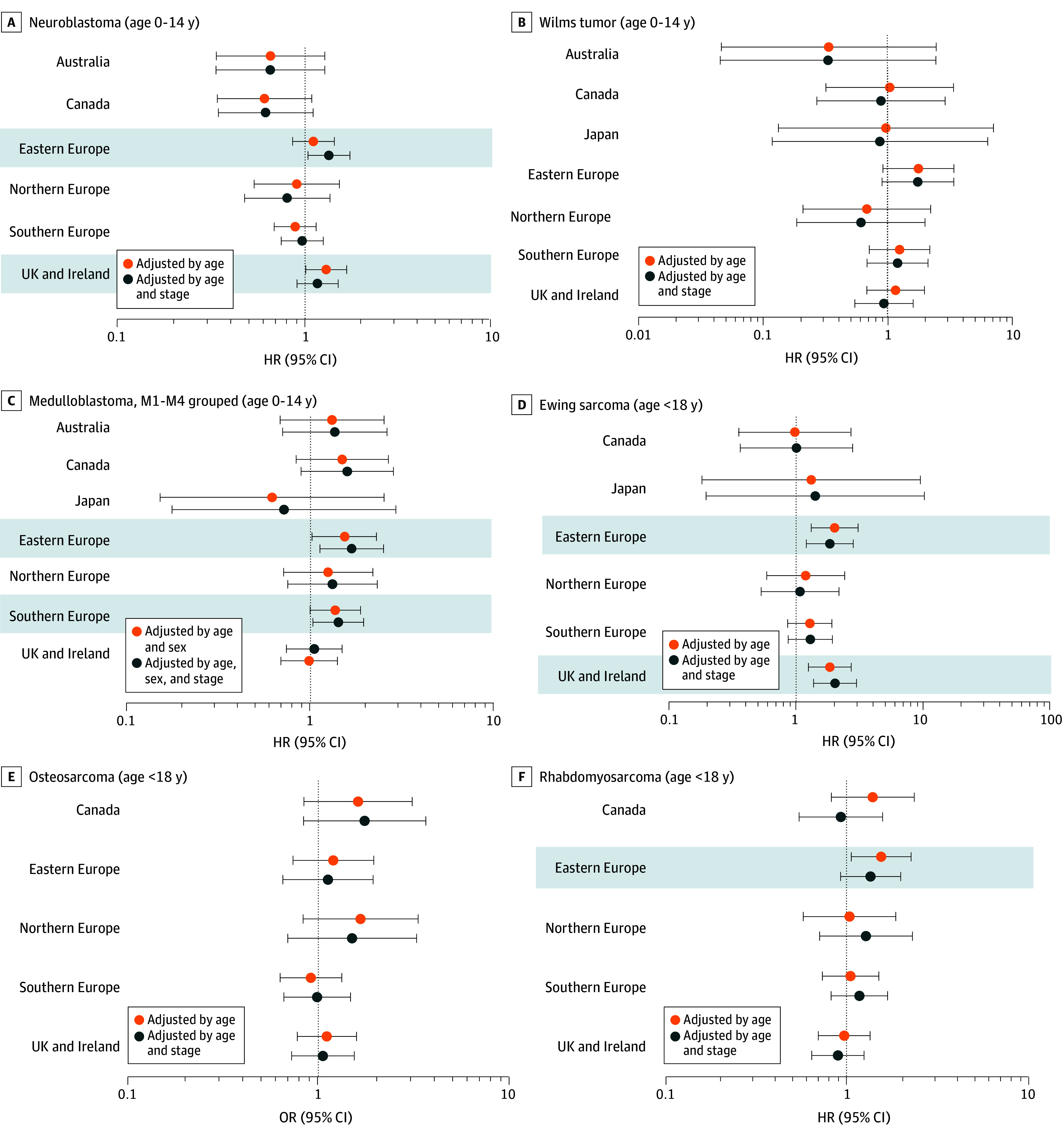
Tumor-Specific Hazard Ratios (HRs) or Odds Ratios (ORs) From Models With and Without Stage Adjustment, by Geographical Area Central Europe was the reference category, and areas with significant differences from Central Europe are shaded. The vertical line at 1 represents the null value for the effect estimates, indicating no difference in the 3-year risk of death compared with the reference category, after adjustment for covariates. Points to the right of this line indicate a higher risk relative to Central Europe, while points to the left indicate a lower risk. HRs are reported for all cancers except osteosarcoma, where the proportional hazards assumption was violated; therefore, ORs estimated using an inverse probability censoring-weighted logistic model are shown. Horizontal bars represent the 95% CI for each estimate.

Comparisons of overall survival between areas are presented as forest plots for each tumor type (Figure 2). HRs are reported for each area or country compared with Central Europe (Austria, Belgium, France, Germany, Switzerland, and the Netherlands) except for osteosarcoma, for which ORs are reported (eTable 5 in Supplement 1). For each tumor type, 2 models were fitted: one including geographical area and age group only (Figure 2, labeled “adjusted by age”) and another including area, age group, and stage (Figure 2, labeled “adjusted by age and stage”). Including sex as a covariate did not improve model fit for any cancer type except medulloblastoma. Consequently, sex was excluded from all models except medulloblastoma, for which Figure 2 presents estimates adjusted for sex. For other cancers, coefficients with and without sex differed only at the second decimal place and did not materially affect the associations under investigation (data not shown).

Three main patterns were observed. In the first pattern, for Wilms tumor and osteosarcoma, there were no significant survival differences between areas. The risk of death remained similar when adjusted by age group only or by age group and stage (Figure 2).

The second pattern was observed for Ewing sarcoma and medulloblastoma. For Ewing sarcoma, 2 areas—Eastern Europe and UK and Ireland—demonstrated a significantly higher risk of death compared with Central Europe. This higher risk persisted after additional adjustment for stage; in Eastern Europe, age-adjusted HR was 2.04 (95% CI, 1.34-3.12), and the age- and stage-adjusted HR was 1.87 (95% CI, 1.22-2.86). In the UK and Ireland, the age-adjusted HR was 1.87 (95% CI, 1.27-2.75), and the age- and stage-adjusted HR was 2.06 (95% CI, 1.39-3.04) (Figure 2; eTable 5 in Supplement 1). In both areas, the survival differences were only significant for the metastatic group (Figure 1). For medulloblastoma (with all metastatic patients grouped), the HRs adjusted for sex and age through stratification were significantly higher in Eastern Europe (HR, 1.54; 95% CI, 1.03-2.28) when compared with Central Europe. After adjusting also for stage, these associations remained (HR, 1.68; 95% CI, 1.13-2.49).

The third pattern, in which adjustment for stage changed the significance of the HRs, was seen in 3 tumor types. For neuroblastoma, the UK and Ireland area showed a statistically significantly higher risk of death in the age-adjusted model, with an HR of 1.31 (95% CI, 1.02-1.69), decreasing to 1.18 (95% CI, 0.91-1.52) after adjustment for tier 1 stage, so that the difference was no longer statistically significant (Figure 2; eTable 5 in Supplement 1). By contrast, Eastern Europe had a not statistically significant age-adjusted HR (1.12; 95% CI, 0.87-1.45), which changed to a statistically significant HR (1.36; 95% CI, 1.05-1.76) when adjusting also by stage. An additional analysis, excluding Germany and using tier 2 stage, found that the differences observed across geographical areas lost statistical significance; however, the magnitude, behavior, and direction of the HRs remained similar to those in the model analyzing tier 1 stage (eTable 6 in Supplement 1). For rhabdomyosarcoma, Eastern Europe showed a higher risk of death in the age-adjusted model (HR, 1.53; 95% CI, 1.05-2.23). After adjusting for stage, there was no association (HR, 1.34; 95% CI, 0.91-1.96), suggesting that part of the excess risk-observed difference may be explained by differences in stage distribution. For medulloblastoma, the HR adjusted for age and sex in Southern Europe was 1.36 (95% CI, 0.99-1.87) and became significantly higher compared with Central Europe after adjusting by stage (with all metastatic patients grouped) (HR, 1.42; 95% CI, 1.03-1.94).

The sensitivity analysis performed to quantify the possible impact of a high proportion of cases with missing stage for medulloblastoma for the UK and Ireland reported a slight increase in the probability of death when re-allocating the missing stage cases in the different scenarios (HR increased from 1.00 to 1.12). However, all HRs remained not statistically significant (data not shown).

## Discussion

The BENCHISTA project represents a successful international collaborative effort to apply the consensus TG for childhood cancer with CRs able to provide the necessary data, using cases diagnosed from 2014 to 2017 with sufficient follow-up for survival. To our knowledge, this is the first time that population-level 3-year survival by stage for 6 pediatric solid tumors has been compared across geographical areas. As expected from clinical trials, we found stage at diagnosis to be a strong prognostic factor across all 6 tumor types (eFigure 1 in Supplement 1). We found significant variation in 3-year overall survival between some geographical areas and countries for 4 tumors (Figures 1 and 2). For neuroblastoma (UK and Ireland) and rhabdomyosarcoma (Eastern Europe), variation in stage distribution at diagnosis was a crucial factor associated with geographic survival variation, with higher proportions of metastatic and advanced stage cases compared with the Central Europe reference group accounting for the observed survival differences. Conversely, in Eastern Europe, stage adjustment of survival analysis worsened the survival gap compared with Central Europe. The higher proportion of localized cases (29%) in Eastern Europe vs 18% in Central Europe may be explained by underdiagnosis of metastatic disease in Eastern Europe, due to lower use of the most sensitive staging investigation (iodine meta-iodobenzylguanidine radioisotope scan) to detect bone marrow metastases.^21^

A similar pattern, in which stage adjustment revealed a significantly increased risk of death compared with Central Europe, was seen for medulloblastoma in Southern Europe. Here, we hypothesize that cases are understaged due to the absence or delayed examination of CSF, which may lead to undertreatment. An Italian study of 96 medulloblastoma cases reported similar findings.^26^ Access to CSF results in clinical records can be challenging for CRs, given that this staging investigation should be performed at least 14 days after surgical resection of the primary tumor.

By contrast, adjusting for stage had little effect on the estimated risk of death relative to Central Europe for Ewing sarcoma in Eastern Europe and in the UK and Ireland or for medulloblastoma in Eastern Europe. For Ewing sarcoma, this implicates other factors, such as variation in treatment. Notably, these geographical survival differences were most pronounced in cases with metastatic disease. In contrast, survival for patients with localized disease did not differ significantly from Central Europe (Figure 1; eTable 4 in Supplement 1). At the time of diagnosis of patients included in this project, 2 international clinical trials were recruiting patients with Ewing sarcoma widely across Europe.^27,28^ While these provided standardized systemic treatment approaches for most patients, there is a lack of consensus for patients with bone and bone marrow metastases or widely disseminated disease, who have significantly worse survival outcomes.^29^ Differences in the sites or extent of metastases in this patient cohort or approaches to their management between geographical regions may be an important contributing factor to survival outcomes.

We did not find any significant geographical variation in 3-year overall survival for Wilms tumor and osteosarcoma (Figure 2). For Wilms tumor, this may reflect the low mortality observed in this generally favorable-prognosis tumor and the widespread use of similar treatment guidelines in most countries contributing cases.^30^ For osteosarcoma, although no multinational European trial has been available since the European and American Osteosarcoma Studies (EURAMOS trial),^31^ which closed in 2012, standardized treatment strategies have been used in most countries and demonstrate comparable efficacy.^32^

Childhood cancer incidence rates vary across geographic regions. While marked global differences have been observed (eg, Japanese vs White populations), within Europe these variations are smaller but still relevant, with Eastern and occasionally Northern European countries showing lower rates—although differences are often not statistically significant due to disease rarity.^33^ Known risk factors explain little of this variation; a plausible explanation may be differences in diagnostic intensity, where countries with higher incidence might also detect more early-stage cases, potentially improving survival. This mechanism highlights how incidence can indirectly influence survival through stage distribution. Our analyses are adjusted for both age and stage, but further exploration of these factors—including diagnostic lead time and tumor biology—will be addressed in the next phase of the project, when more complete data and longer follow-up are available.

A major strength of the BENCHISTA study is the use of population-based CR data, assessing all incident cases within the time period, with a high level of completeness of tumor stage using the TG, and a high proportion with complete follow-up for vital status up to three years. CRs provide the foundation for structured data collection according to standard international rules and ensure complete coverage, reduce likelihood of bias, and allow accurate comparisons across regions and over time.

### Limitations

This study has limitations, including a relatively small number of cases and events and only 3-year follow-up for survival, thus limiting the statistical power to detect survival variation and the need for grouping into geographical areas. However, these limitations were unavoidable considering the rarity of pediatric cancers and because CRs find it easier to access the information needed for TG staging on more recently diagnosed cases.

Some registries could not provide data on all 6 tumors due to difficulties accessing data held in clinical registries; this was the case for Germany (Ewing sarcoma and rhabdomyosarcoma). Despite European data protection regulations aiming to facilitate research, not all European countries were able to participate in this project due to national interpretation of rules preventing patient-level data sharing, even when data were pseudonymized.

Furthermore, differences in HRs between models adjusted for nested models should be interpreted with caution, as these estimates are noncollapsible. Changes in point estimates may reflect not only the effect of stage but also the inherent property of noncollapsibility and potential mediation. Further investigation is needed to develop a causal framework, as the association between country of residence and overall survival may also be mediated by factors such as socioeconomic status, health care accessibility (eg, urban vs rural), availability of treatment strategies, genetic predisposition proxies (eg, nationality or race), and comorbidities.

## Conclusions

In this population-based cohort study, variations in stage at diagnosis were associated with the observed disparities in overall survival between geographical regions for some tumors. These variations are likely attributable to delays in diagnosis or stage migration influenced by differences in sensitivity of staging investigations used. The BENCHISTA project highlights the need to improve early diagnosis and to achieve standardized and universally accessible diagnostic investigations for staging. Where stage variation is not associated with geographical survival differences, other factors should be investigated and are being pursued in BENCHISTA phase 2. These include NSPs commonly used in clinical risk stratification treatment modalities received and cause of death.

## Notes

Supplement 1. **eTable 1.** Contributing Years, Number of Cases, and Quality Indicators to Evaluate the Follow-Up by Country **eFigure 1.** Map of Participating Countries Divided by Geographical Areas **eFigure 2.** Inclusion Criteria Flow Chart **eTable 2.** Three-Year Overall Survival (OS) for the BENCHISTA European Countries **eTable 3.** Three-Year Overall Survival (OS) and 95% Confidence Interval (CI) by Tumor Type and Stage **eFigure 3.** Overall Survival (OS) by Stage for Each Tumor Type **eTable 4.** Three-Year Overall Survival (OS) and 95% Confidence Intervals (CI) for Each Tumor Type and Geographical Area/BENCHISTA Europe and by Stage **eTable 5.** Multivariable Model Results for Each Tumor Type **eTable 6.** Multivariable Age-Stratified Cox Model for Neuroblastoma Using Tier 2 Data and Excluding Germany **eAppendix 1.** The BENCHISTA Project Working Group **eAppendix 2.** Toronto Childhood Cancer Stage Guidelines—Stage Definition by Tumur Type as Used in the BENCHISTA Project
eReferences.

Supplement 2.
Nonauthor Collaborators

Supplement 3.
Data Sharing Statement
