# Supplementary material for: Stage at Diagnosis and International Survival Variation in Childhood Tumors in the BENCHISTA Study
Source: JAMA Netw Open. 2026 Feb 9;9(2):e2556747. doi: 10.1001/jamanetworkopen.2025.56747 (PMC12887745; doi:10.1001/jamanetworkopen.2025.56747)
Supplement: Supplement 1. — eTable 1. Contributing Years, Number of Cases, and Quality Indicators to Evaluate the Follow-Up by Country eFigure 1. Map of Participating Countries Divided by Geographical Areas eFigure 2. Inclusion Criteria Flow Chart eTable 2. Three-Year Overall Survival (OS) for the BENCHISTA European Countries eTable 3. Three-Year Overall Survival (OS) and 95% Confidence Interval (CI) by Tumor Type and Stage eFigure 3. Overall Survival (OS) by Stage for Each Tumor Type eTable 4. Three-Year Overall Survival (OS) and 95% Confidence Intervals (CI) for Each Tumor Type and Geographical Area/BENCHISTA Europe and by Stage eTable 5. Multivariable Model Results for Each Tumor Type eTable 6. Multivariable Age-Stratified Cox Model for Neuroblastoma Using Tier 2 Data and Excluding Germany eAppendix 1. The BENCHISTA Project Working Group eAppendix 2. Toronto Childhood Cancer Stage Guidelines—Stage Definition by Tumur Type as Used in the BENCHISTA Project eReferences. [file jamanetwopen-e2556747-s001.pdf]

Supplemental Online Content

Botta L, Didonè F, Lopez-Cortes A, et al; The BENCHISTA Project Working Group. Stage at diagnosis and international survival variation in childhood tumors in the BENCHISTA study. *JAMA Netw Open.* 2026;9(2):e2556747. doi:10.1001/jamanetworkopen.2025.56747

eTable 1. Contributing Years, Number of Cases, and Quality Indicators to Evaluate the Follow-Up by Country

eFigure 1. Map of Participating Countries Divided by Geographical Areas

eFigure 2. Inclusion Criteria Flow Chart

eTable 2. Three-Year Overall Survival (OS) for the BENCHISTA European Countries

eTable 3. Three-Year Overall Survival (OS) and 95% Confidence Interval (CI) by Tumor Type and Stage

eFigure 3. Overall Survival (OS) by Stage for Each Tumor Type

eTable 4. Three-Year Overall Survival (OS) and 95% Confidence Intervals (CI) for Each Tumor Type and Geographical Area/BENCHISTA Europe and by Stage

eTable 5. Multivariable Model Results for Each Tumor Type

eTable 6. Multivariable Age-Stratified Cox Model for Neuroblastoma Using Tier 2 Data and Excluding Germany

eReferences.

This supplemental material has been provided by the authors to give readers additional information about their work.

© 2026 Botta L et al. *JAMA Network Open*.

eTable 1. Contributing Years, Number of Cases, and Quality Indicators to Evaluate the Follow-Up by Country

Area	Country	Contributing years	%DCO	Total cases (18+ excluded) §	Cases included	Quality indicators											
						Neuro_blastoma		Wilms tumour		Medullo_blastoma		Osteo_sarcoma		Ewing sarcoma		Rhabdomyo_sarcoma	
						N/D	%	N/D	%	N/D	%	N/D	%	N/D	%	N/D	%
Central Europe	Austria	2015-2017	0.1	157	157	0/41	0.0	0/28	0.0	0/17	0.0	0/23	0.0	0/20	0.0	0/28	0.0
	Belgium	2015-2017	0	195	195	2/52	3.9	0/45	0.0	0/24	0.0	0/28	0.0	0/22	0.0	0/24	0.0
	France	2014-2016	0	1,428	1,428	0/391	0.0	0/232	0.0	0/203	0.0	0/208	0.0	0/192	0.0	0/202	0.0
	Germany	2014-2017	0	1,307	1,307	17/453	3.8	6/383	1.6	9/235	3.8	2/236	0.8	NA	..	NA	..
	Netherlands	2014-2017	NA	437	437	0/94	0.0	0/82	0.0	0/75	0.0	1/52	1.9	0/61	0.0	0/73	0.0
	Switzerland	2015-2017	0.3	167	167	0/52	0.0	0/27	0.0	0/24	0.0	0/25	0.0	0/12	0.0	0/27	0.0
Northern Europe	Denmark	2014-2017	0	57	57	0/26	0.0	0/18	0.0	0/13	0.0	NE	..	NE	..	NE	..
	Norway	2014-2016	0.2	88	88	0/18	0.0	1/21	4.8	0/9	0.0	0/9	0.0	0/12	0.0	0/19	0.0
	Sweden	2014-2017	0	264	264	0/58	0.0	0/63	0.0	0/40	0.0	0/34	0.0	0/31	0.0	0/38	0.0
Eastern Europe	Bulgaria	2014-2017	5.6	135	135	0/42	0.0	0/27	0.0	0/15	0.0	0/5	0.0	0/25	0.0	0/21	0.0
	Czechia	2014-2017	0	224	224	2/55	3.6	1/39	2.6	0/42	0.0	0/26	0.0	0/24	0.0	0/38	0.0
	Estonia	2014-2017	0	30	30	0/9	0.0	0/9	0.0	0/4	0.0	0/1	0.0	0/3	0.0	0/4	0.0
	Hungary	2014-2016	0	211	211	1/64	1.6	0/41	0.0	0/27	0.0	0/28	0.0	0/26	0.0	1/25	4.0

© 2026 Botta L et al. *JAMA Network Open*.

	Poland	2014-2016	NA	685	260	14/260	5.4	23/141	16.3	15/84	17.9	12/62	19.4	14/62	22.6	13/76	17.1
	Romania	2014-2016	0	236	236	0/61	0.0	0/40	0.0	0/32	0.0	0/38	0.0	0/29	0.0	1/36	2.8
Southern Europe	Greece	2014-2016	0	122	122	2/54	3.7	0/43	0.0	3/25	12.0	NE	..	NE	..	NE	..
	Italy[*1]	2014-2017	0.1	807	807	3/240	1.3	5/124	4.0	1/100	1.0	0/127	0.0	1/107	0.9	1/109	0.9
	Malta	2014-2017	1.2	6	6	0/1	0.0	0/3	0.0	0/2	0.0	0/0	..	0/0	..	0/0	..
	Portugal	2014-2017	0.1	206	206	0/59	0.0	0/38	0.0	0/28	0.0	0/25	0.0	0/30	0.0	0/26	0.0
	Slovenia	2015-2017	0	36	36	0/8	0.0	0/4	0.0	0/3	0.0	0/5	0.0	0/8	0.0	0/8	0.0
	Spain[2]	2014-2017	0.3	641	641	4/227	1.8	1/131	0.8	3/96	3.1	1/64	1.0	0/63	0.0	0/60	0.0
UK and Ireland	Northern Ireland	2014-2017	0.7	46	46	0/10	0.0	0/17	0.0	0/5	0.0	0/2	0.0	0/6	0.0	0/6	0.0
	Ireland	2014-2017	0.3	137	137	0/34	0.0	0/33	0.0	0/24	0.0	0/25	0.0	0/6	0.0	1/15	6.7
	England	2014-2017	0.1	1,420	1,420	1/335	0.3	1/338	0.3	1/186	0.5	1/185	0.5	1/133	0.8	2/243	0.8
	Scotland	2014-2016	0.2	91	91	0/23	0.0	1/20	5.0	0/13	0.0	0/12	0.0	0/10	0.0	0/13	0.0
	Wales	2014-2017	0	75	75	0/14	0.0	0/17	0.0	0/10	0.0	0/6	0.0	0/7	0.0	0/21	0.0
Extra EU	Australia[*3]	2014-2017	0,3	186	186	0/76	0.0	0/66	0.0	0/44	0.0	NE	..	NE	..	NE	..
	Brazil[*4]	2014-2017	7.4	463	463	11/65	16.9	30/126	23.8	11/69	15.9	15/90	16.7	9/41	22.0	16/72	22.2
	Canada[*5]	2014-2017	0.1	358	358	1/115	0.9	1/63	1.6	0/50	0.0	2/48	4.2	0/24	0.0	2/58	3.4
	Japan[*6]	2014-2016	0.8	151	151	10/68	14.7	2/26	7.7	2/19	10.5	0/12	0.0	0/5	0.0	3/21	14.3
BENCHISTA		2014-2017	0.7	10,366	9,941	68/3,005	2.3	72/2,245	3.2	45/1,518	3.0	34/1,376	2.3	26/959	2.5	40/1,263	2.9

© 2026 Botta L et al. *JAMA Network Open*.

Footnote:

Quality indicators: % of Death certificate only cases (DCO); ratio between N (number of cases lost to follow-up, or vital status or follow-up time unknown) and D (overall number of cases).
Criteria for exclusion: A country was not included in the cancer-specific analysis if the quality indicator N/D > 12%. These countries are highlighted by a black border around the corresponding cell.
§ All cases 0-14 years old for neuroblastoma, medulloblastoma and Wilms tumour and only cases <18 years old for sarcomas to include also some adolescent but ensuring a common age range denominator, with CR selection: excluding Australia, Greece, Spanish Registry of Childhood Tumors (RETI-SEHOP) (Spain), and Denmark as they routinely collected only up to 0-14 years old.
* Countries with partial national coverage.
NA = Data was not made available to the project.
NE = Not eligible as they routinely collected only up to 0-14.
1=CR of Milano, Basilicata, Bergamo, Campania, Catania Messina Enna, Emilia Romagna, Insubria, Liguria, Mantova e Cremona, Marche, Palermo, Ragusa, Sassari, Siracusa, Toscana, Trapani, Umbria, Veneto, Brianza, Friuli Venezia Giulia, Piemonte, Puglia, Trento, Genova, Nuoro.
2=CR of Basque country, Comunidad valenciana, Girona, Granada, Murcia, Navarra, Madrid, RETI SEHOP, Tarragona.
3= Victorian Cancer Registry, Queensland Cancer Registry and Northern Territory Cancer Registry.
4=CRs of Aracaju, Belém, Belo Horizonte, Campinas, Curitiba, Distrito Federal, Barretos,Fortaleza, Jau, João Pessoa, Mato Grosso and Recife.
5=CR of Ontario (Pediatric Oncology Group of Ontario).
6= 62% of the cases in Tokyo and Osaka (1).

© 2026 Botta L et al. *JAMA Network Open*.

eFigure 1. Map of Participating Countries Divided by Geographical Areas

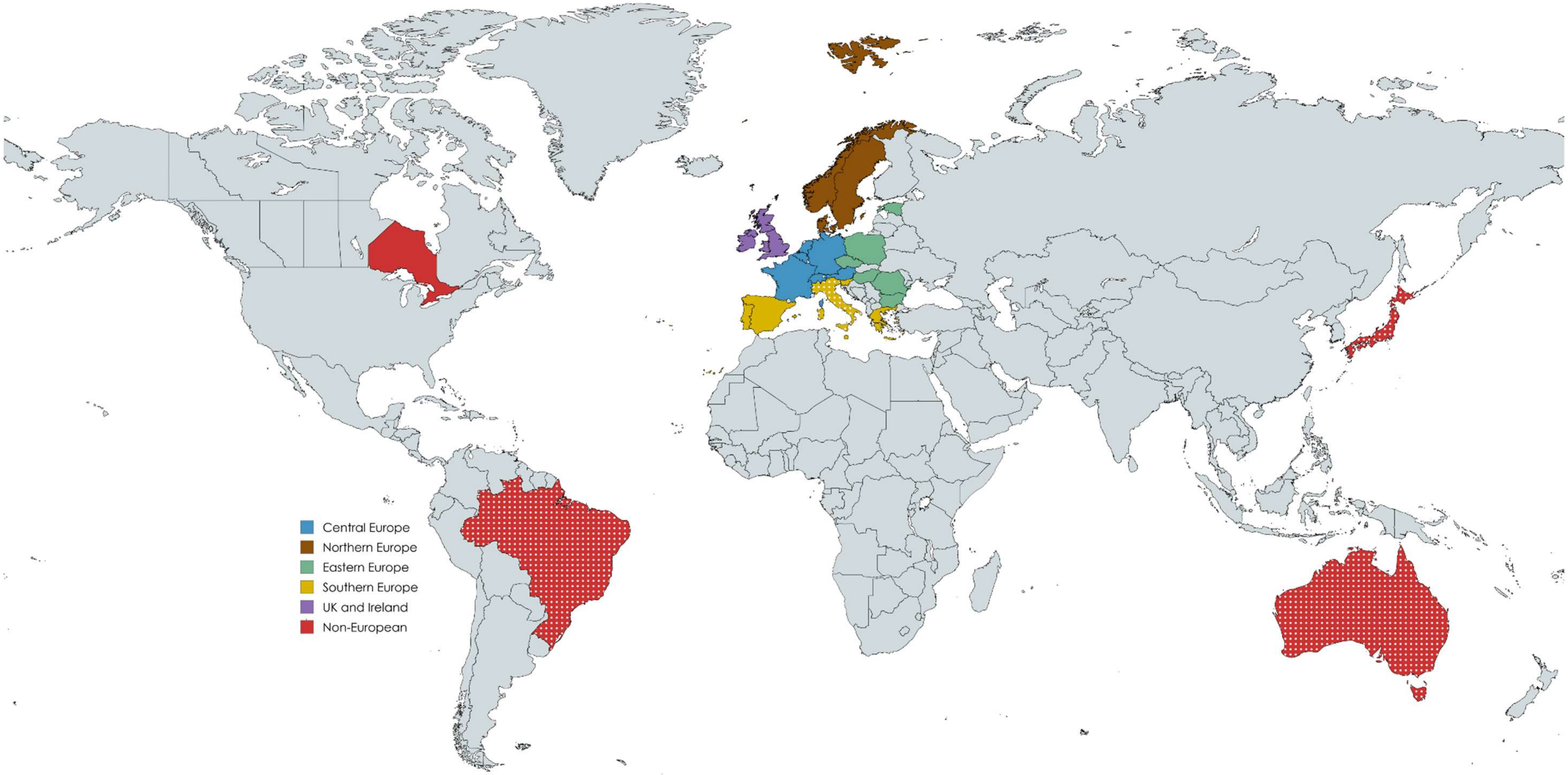

Dotted countries= Not fully geographically covered.

© 2026 Botta L et al. *JAMA Network Open*.

eFigure 2. Inclusion Criteria Flow Chart

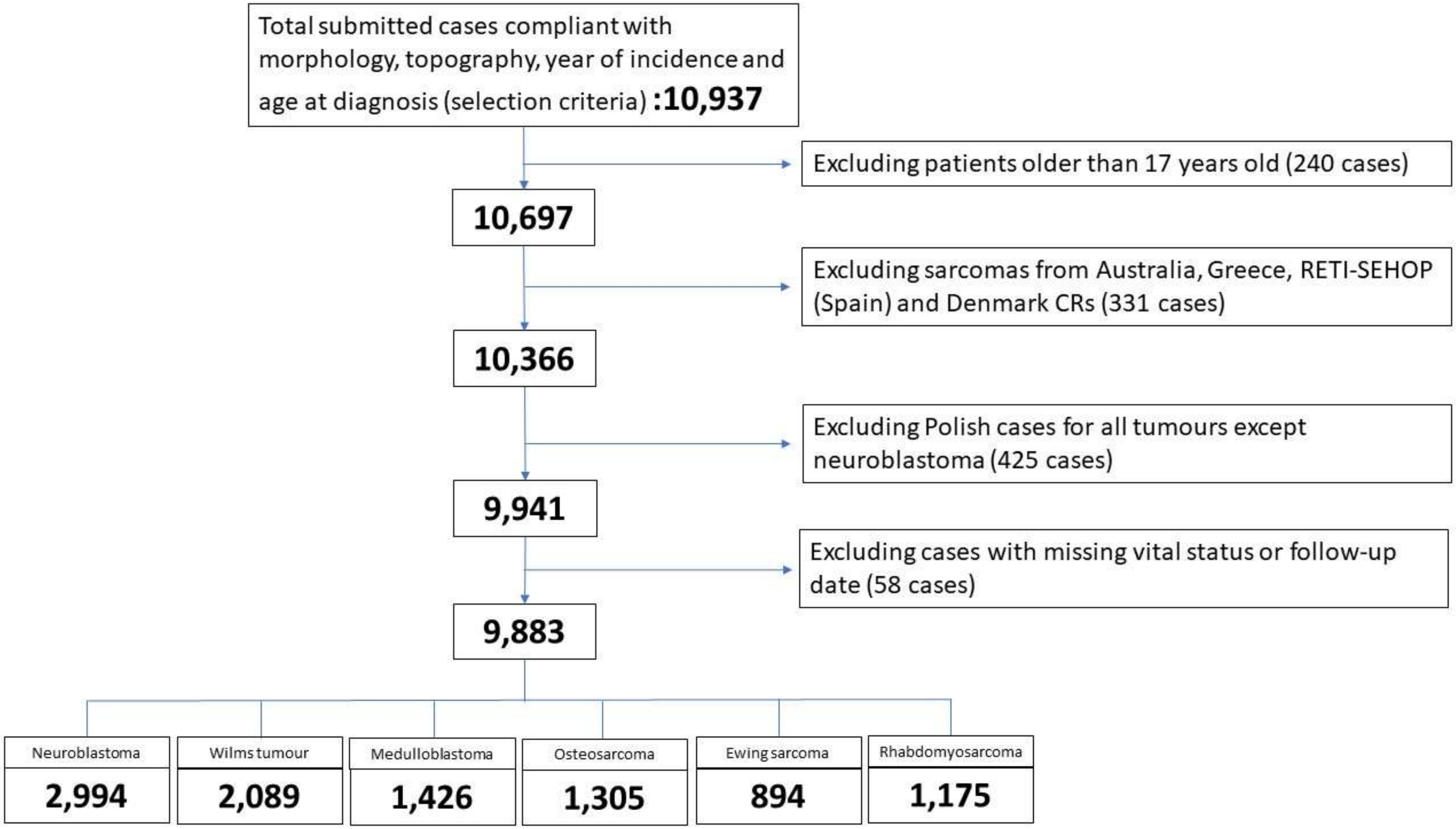

© 2026 Botta L et al. *JAMA Network Open*.

eTable 2. Three-year Overall Survival (OS) for the BENCHISTA European Countries

Type of tumour	BENCHISTA Europe			
	N	Three-year OS	95% confidence interval	
Neuroblastoma	2,681	83%	81%	84%
Wilms tumour	1,817	95%	94%	96%
Medulloblastoma	1,248	79%	77%	81%
Osteosarcoma*	1,163	75%	73%	77%
Ewing sarcoma*	825	78%	75%	80%
Rhabdomyosarcoma*	1,032	77%	74%	79%

Footnote:
Poland has been excluded from all the tumour types except for neuroblastoma. Data on Ewing sarcoma and rhabdomyosarcoma cases from Germany was not made available to the project.
*Sarcomas are calculated including only cases 0-17 years old (see eTable 1 for country selection).

© 2026 Botta L et al. *JAMA Network Open*.

eTable 3. Three-Year Overall Survival (OS) and 95% Confidence Interval (CI) by Tumor Type and Stage

Type of tumour	Stage	Number of cases	Three-year OS	95% CI	
Neuroblastoma (Tier 1)	L	671	98%	97%	99%
	LR	697	92%	90%	94%
	M	1,092	67%	64%	70%
	MS	270	87%	82%	90%
	X	142	89%	82%	93%
Wilms tumour	y-I/I	783	99%	98%	99%
	y-II/II	355	97%	95%	99%
	y-III/III	378	94%	91%	96%
	IV	346	87%	83%	90%
	X	116	92%	85%	96%
Medulloblastoma	M0	791	85%	82%	87%
	M1	86	74%	64%	82%
	M2	99	80%	70%	86%
	M3	242	64%	58%	70%
	M4	4	25%	1%	67%
	X	143	77%	69%	83%
Osteosarcoma[a]	L	850	82%	80%	85%
	M	265	48%	42%	54%
	X	109	75%	65%	82%
Ewing sarcoma[a]	L	559	87%	84%	90%
	M	268	55%	49%	60%
	X	27	89%	69%	96%
Rhabdomyosarcoma[a]	I	324	95%	92%	97%
	II	119	87%	80%	92%
	III	266	81%	76%	85%
	IV	280	45%	39%	50%
	X	105	77%	69%	84%

Footnote:
Countries excluded due to potential follow-up issues: Brazil (all tumours), Japan (neuroblastoma and rhabdomyosarcoma), and Poland (all cancers except neuroblastoma). Data on Ewing sarcoma and rhabdomyosarcoma cases from Germany was not made available to the project.
a=Sarcomas are calculated including only cases 0-17 years old (see eTable 1 for country selection).

© 2026 Botta L et al. *JAMA Network Open*.

eFigure 3. Overall Survival (OS) by Stage for Each Tumor Type

Neuroblastoma (age 0-14 y)

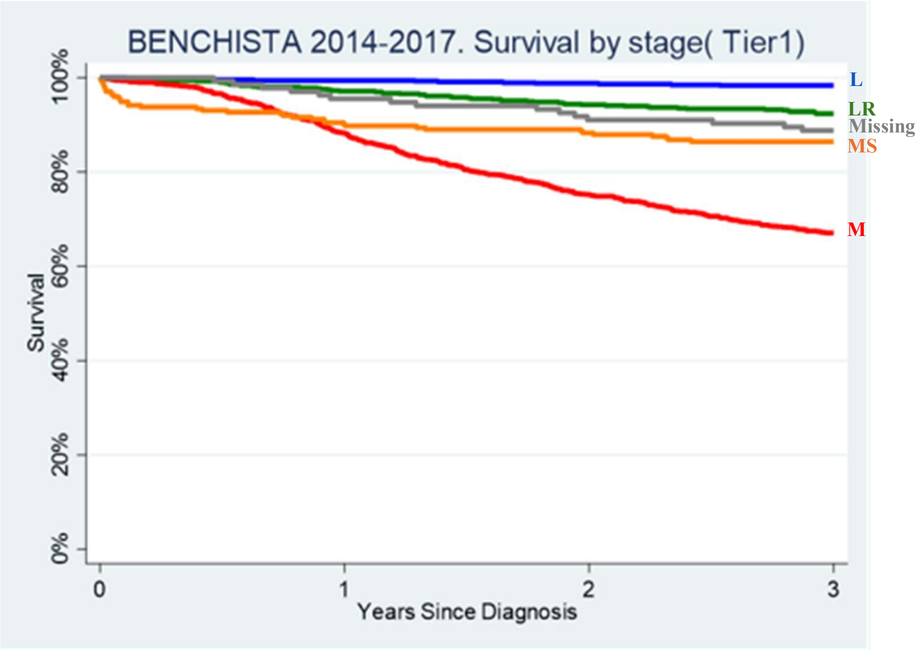

L: Localised.
LR: Locoregional.
MS: Metastatic disease confined to skin, liver, and/or bone marrow in a patient <18 months.
M: Metastatic.

Number of people at risk

Stage	Time in years since diagnosis			
	0	1	2	3
L	669	660	648	641
LR	696	675	653	632
M	1094	964	821	726
MS	267	241	236	230
Missing	137	128	121	118

Wilms tumour (age 0-14 y)

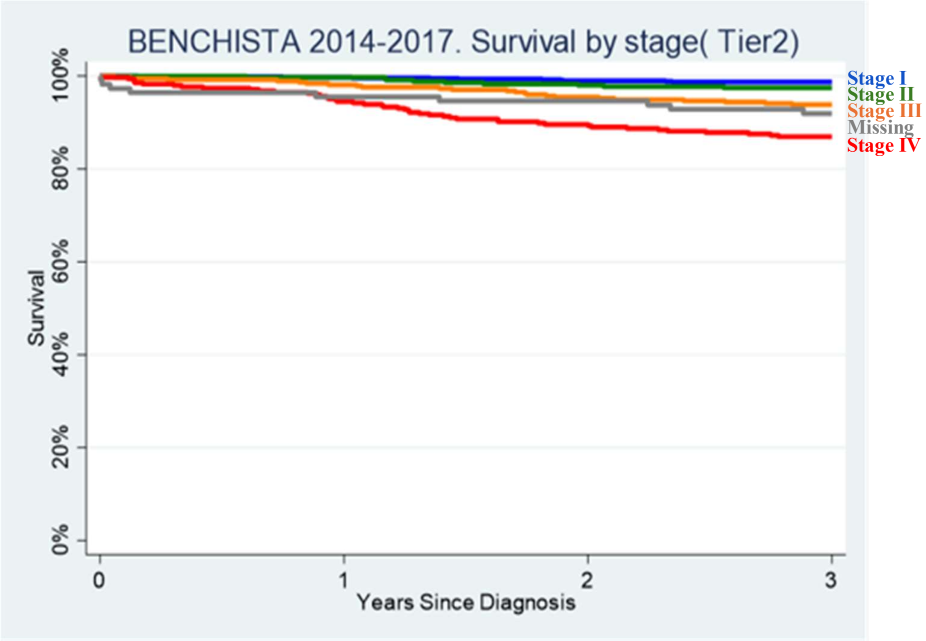

y=Tumour staged after preoperative chemotherapy.

y-I/I=Tumour is limited to the kidney and completely excised.
y-II/II=Tumour extends beyond kidney but completely resected.
y-III/III=Incomplete excision of the tumour (gross or microscopic extension beyond the resection margins) or with residual tumour or non-haematogenous metastases confined to abdomen.
IV=Haematogenous metastases or spread beyond abdomen at diagnosis.

Number of people at risk

Stage	Time in years since diagnosis			
	0	1	2	3
I	781	777	769	767
II	355	354	348	343
III	377	368	357	351
IV	346	327	308	297
Missing	112	107	106	102

© 2026 Botta L et al. *JAMA Network Open*.

Medulloblastoma (age 0-14 y)

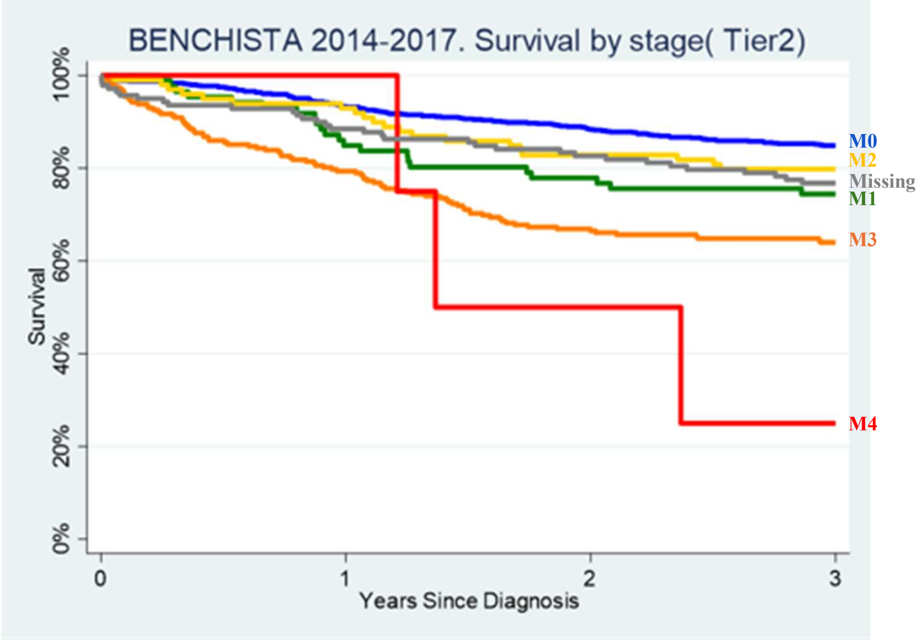

M0: Localised disease.
M1: Tumour cells in CSF.
M2: Visible metastasis in brain.
M3: Visible metastases in spine or in cervicomedullary (junction).
M4: Visible metastasis outside of the central nervous system.

Number of people at risk

Stage	Time in years since diagnosis			
	0	1	2	3
MO	790	734	695	662
M1	86	73	67	63
M2	99	92	82	77
M3	242	192	160	153
M4	4	4	2	1
Missing	140	122	113	105

Osteosarcoma (age <18 y)

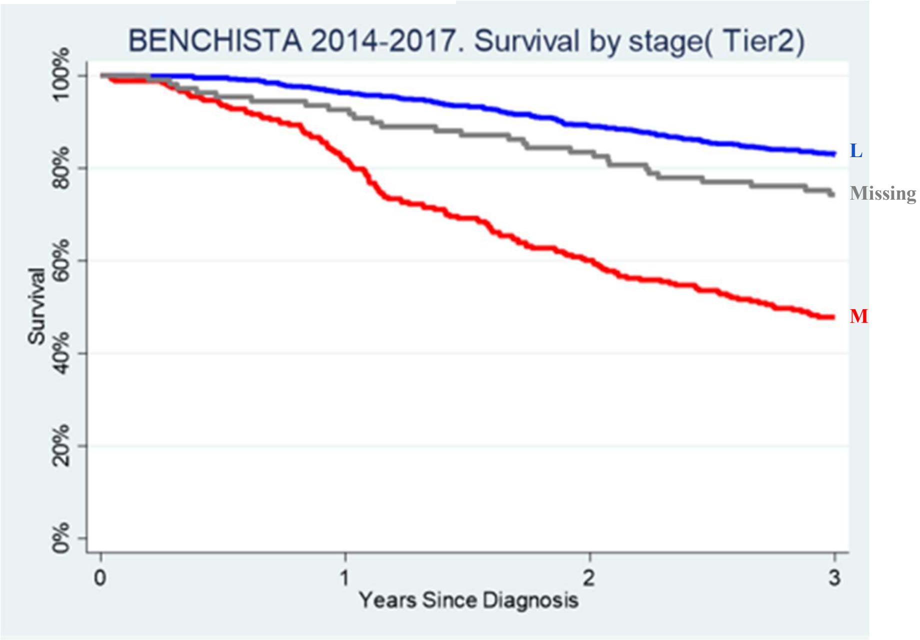

L: Localised.
M: Metastatic.

Number of people at risk

Stage	Time in years since diagnosis			
	0	1	2	3
L	850	818	754	698
M	265	215	158	125
Missing	109	101	91	81

© 2026 Botta L et al. *JAMA Network Open*.

Ewing sarcoma (age <18 y)

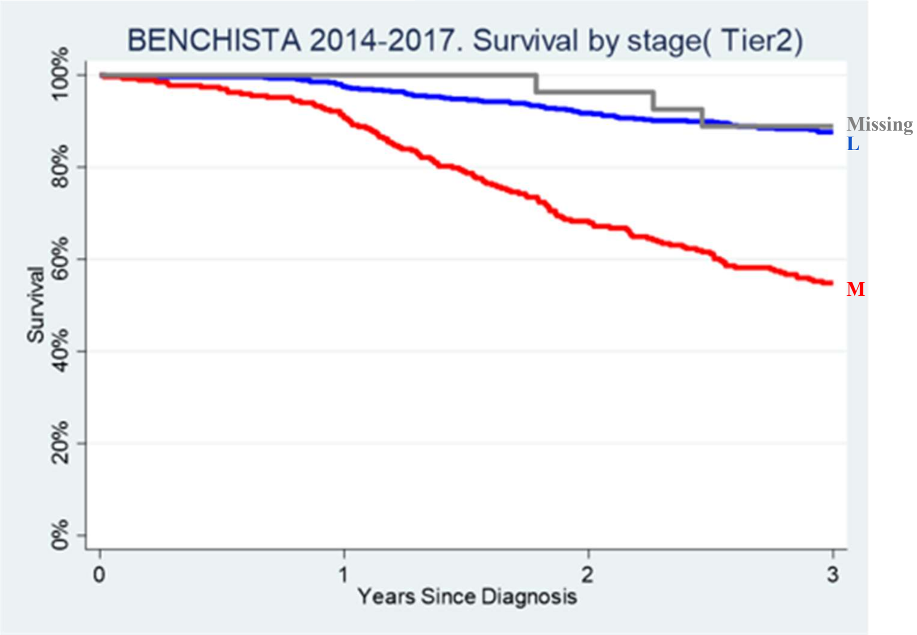

L: Localised.
M: Metastatic.

Number of people at risk

Stage	Time in years since diagnosis			
	0	1	2	3
L	560	544	511	487
M	269	243	183	146
Missing	27	27	26	24

Rhabdomyosarcoma (age <18 y)

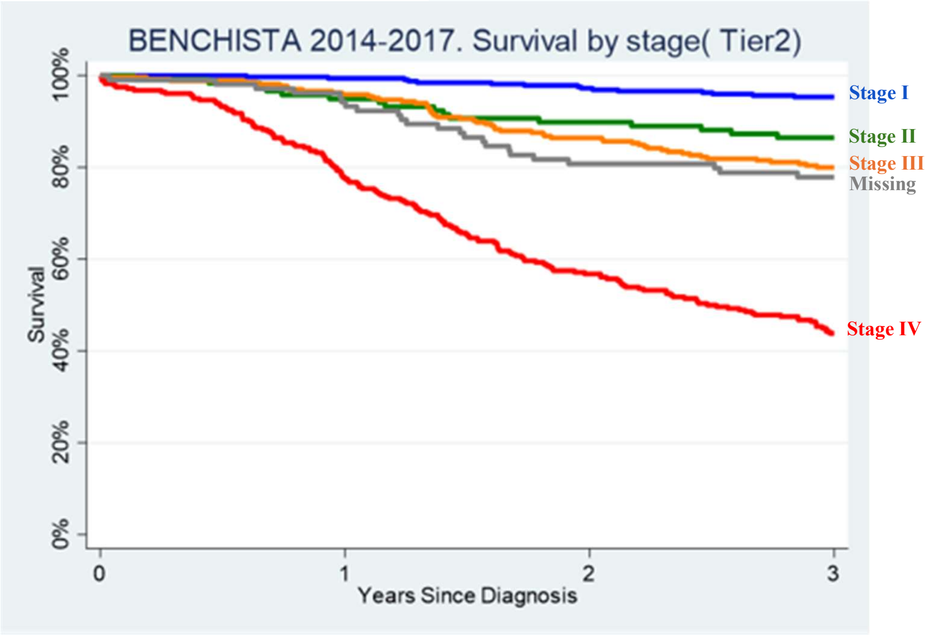

I: Favourable site, any tumour size, any involvement of regional lymph nodes.
II: Unfavourable site, tumour ≤ 5cm in greatest dimension, no regional lymph nodes involved.
III: Unfavourable site, tumour ≤ 5cm in greatest dimension, with regional lymph nodes involvement but no distant metastasis, or unfavourable site and tumour > 5cm in greatest dimension with any node involvement and no distant metastasis.
IV: Tumour has spread to distant metastatic sites.

Number of people at risk

Stage	Time in years since diagnosis			
	0	1	2	3
I	324	322	313	306
II	119	112	106	102
III	266	254	229	212
IV	280	217	159	121
Missing	105	98	84	81

© 2026 Botta L et al. *JAMA Network Open*.

eTable 4. Three-Year Overall Survival (OS) and 95% Confidence Intervals (CI) for Each Tumor Type and Geographical Area/BENCHISTA Europe and by Stage

	Neuroblastoma Tier1 (age 0-14 y)																							
	L				LR				M				MS				X				Overall			
	No.	Three-year OS	95%CI		No.	Three-year OS	95%CI		No.	Three-year OS	95%CI		No.	Three-year OS	95%CI		No.	Three-year OS	95%CI		No.	Three-year OS	95%CI	
Central Europe	195	99%	96%	100%	300	93%	89%	95%	415	68%	63%	72%	89	89%	80%	94%	84	90%	80%	95%	1,083	84%	81%	86%
Eastern Europe	143	99%	94%	100%	145	92%	87%	96%	154	59%	51%	66%	45	78%	63%	87%	2	100%	.	.	489	82%	79%	85%
Northern Europe	20	100%	.	.	20	100%	.	.	47	68%	53%	79%	15	100%	.	.	0	.	.	.	102	85%	77%	91%
Southern Europe	182	97%	94%	99%	128	93%	87%	96%	201	70%	63%	75%	61	87%	75%	93%	13	85%	51%	96%	585	85%	82%	88%
UK and Ireland	77	99%	91%	100%	68	85%	74%	92%	207	65%	58%	71%	34	88%	72%	95%	29	89%	70%	96%	415	78%	74%	82%
**BENCHISTA Europe**	**617**	**98%**	**97%**	**99%**	**661**	**92%**	**90%**	**94%**	**1,021**	**66%**	**63%**	**69%**	**247**	**87%**	**82%**	**90%**	**128**	**89%**	**82%**	**94%**	**2,674**	**83%**	**81%**	**84%**
Canada	34	100%	.	.	22	95%	72%	99%	41	79%	63%	88%	18	88%	59%	97%	0	.	.	.	115	90%	82%	94%
Australia	17	94%	65%	99%	10	100%	.	.	27	81%	61%	92%	8	88%	39%	98%	14	86%	54%	96%	76	88%	78%	94%
Japan (*)	10	100%	.	.	18	100%	.	.	30	83%	64%	93%	2	100%	.	.	6	100%	.	.	66	92%	81%	96%
Brazil (*)	12	92%	54%	99%	9	56%	20%	80%	34	50%	32%	66%	6	42%	6%	77%	2	50%	1%	91%	63	58%	44%	69%

© 2026 Botta L et al. *JAMA Network Open*.

	Wilms tumour (age 0-14 y)																							
	**y-I/I**				**y-II/II**				**y-III/III**				**IV**				**X**				**Overall**			
	**No.**	**Three-year OS**	**95%CI**		**No.**	**Three-year OS**	**95%CI**		**No.**	**Three-year OS**	**95%CI**		**No.**	**Three-year OS**	**95%CI**		**No.**	**Three-year OS**	**95%CI**		**No.**	**Three-year OS**	**95%CI**	
Central Europe	370	99%	97%	100%	135	98%	93%	99%	91	96%	89%	98%	140	86%	80%	91%	61	92%	81%	96%	797	96%	94%	97%
Eastern Europe	61	97%	88%	99%	35	97%	81%	100%	34	91%	75%	97%	24	79%	56%	91%	2	50%	1%	91%	156	93%	89%	95%
Northern Europe	44	98%	85%	100%	19	95%	68%	99%	21	95%	71%	99%	18	100%	.	.	0	.	.	.	102	97%	91%	99%
Southern Europe	127	98%	94%	100%	63	98%	90%	100%	74	93%	85%	97%	47	81%	66%	90%	27	93%	74%	98%	338	94%	91%	96%
UK and Ireland	138	99%	95%	100%	64	97%	88%	99%	108	92%	85%	96%	93	90%	82%	95%	21	95%	69%	99%	424	95%	92%	97%
**BENCHISTA Europe**	**740**	**99%**	**97%**	**99%**	**316**	**97%**	**95%**	**99%**	**328**	**93%**	**90%**	**96%**	**322**	**87%**	**83%**	**90%**	**111**	**92%**	**85%**	**96%**	**1,817**	**95%**	**94%**	**96%**
Canada	14	100%	.	.	18	94%	67%	99%	18	94%	67%	99%	13	92%	57%	99%	0	.	.	.	63	95%	86%	98%
Australia	20	100%	.	.	14	100%	.	.	23	100%	.	.	NR	.	.	.	NR	.	.	.	66	98%	90%	100%
Japan	8	100%	.	.	5	100%	.	.	7	100%	.	.	4	75%	13%	96%	0	.	.	.	24	96%	74%	99%
Brazil (*)	63	88%	76%	94%	13	82%	45%	95%	7	71%	26%	92%	23	53%	31%	72%	13	73%	38%	91%	119	78%	68%	84%

© 2026 Botta L et al. *JAMA Network Open*.

	Medulloblastoma° (age 0-14 y)																							
	M0				M1				M2				M3				X				Overall			
	No.	Three-year OS	95%CI		No.	Three-year OS	95%CI		No.	Three-year OS	95%CI		No.	Three-year OS	95%CI		No.	Three-year OS	95%CI		No.	Three-year OS	95%CI	
Central Europe	344	87%	82%	90%	42	83%	68%	92%	51	80%	67%	89%	97	68%	58%	76%	43	74%	57%	85%	578	82%	78%	85%
Eastern Europe	78	83%	73%	90%	6	50%	11%	80%	9	67%	28%	88%	20	50%	27%	69%	5	60%	13%	88%	120	74%	67%	80%
Northern Europe	44	75%	59%	85%	5	80%	20%	97%	3	100%	.	.	9	78%	36%	94%	1	100%	.	.	62	77%	65%	86%
Southern Europe	150	82%	75%	87%	11	58%	27%	80%	13	85%	51%	96%	47	62%	46%	74%	30	70%	50%	83%	251	76%	70%	81%
UK and Ireland	101	87%	79%	92%	17	71%	43%	87%	18	72%	46%	87%	47	68%	53%	79%	54	85%	73%	92%	237	81%	75%	85%
**BENCHISTA Europe**	**717**	**85%**	**82%**	**87%**	**81**	**74%**	**63%**	**82%**	**94**	**79%**	**69%**	**86%**	**220**	**65%**	**59%**	**71%**	**133**	**78%**	**69%**	**84%**	**1,248**	**79%**	**77%**	**81%**
Canada	33	88%	71%	95%	1	.	.	.	3	100%	.	.	12	42%	15%	67%	0	.	.	.	50	74%	59%	84%
Australia	25	88%	67%	96%	NR	.	.	.	NR	.	.	.	9	56%	20%	80%	8	63%	23%	86%	44	77%	62%	87%
Japan	14	85%	51%	96%	2	100%	.	.	1	.	.	.	1	100%	.	.	1	100%	.	.	19	89%	62%	97%
Brazil (*)	41	79%	63%	89%	4	50%	6%	84%	6	17%	1%	52%	8	47%	12%	76%	5	.	.	.	65	63%	50%	74%

© 2026 Botta L et al. *JAMA Network Open*.

	Osteosarcoma (age <18 y)															
	L				M				X				Overall			
	No. cases	Three-year OS	95%CI		No. cases	Three-year OS	95%CI		No. cases	Three-year OS	95%CI		No. cases	Three-year OS	95%CI	
Central Europe	373	85%	81%	89%	118	48%	38%	56%	81	74%	63%	82%	572	76%	72%	79%
Eastern Europe	71	76%	64%	84%	27	48%	29%	65%	0	.	.	.	98	78%	71%	84%
Northern Europe	31	74%	55%	86%	12	42%	15%	67%	0	.	.	.	43	71%	57%	81%
Southern Europe	174	83%	77%	88%	41	51%	35%	65%	6	50%	11%	80%	221	74%	68%	79%
UK and Ireland	153	82%	75%	88%	56	46%	33%	59%	20	80%	55%	92%	229	73%	67%	79%
**BENCHISTA Europe**	**802**	**83%**	**80%**	**86%**	**254**	**48%**	**41%**	**54%**	**107**	**74%**	**66%**	**81%**	**1163**	**75%**	**73%**	**77%**
Canada	38	73%	55%	84%	9	44%	14%	72%	1	100%	.	.	48	68%	53%	79%
Japan	10	100%	.	.	1	100%	.	.	1	100%	11%	80%	12	100%	.	.
Brazil (*)	38	73%	56%	85%	36	26%	13%	42%	8	21%	1%	59%	82	49%	38%	60%

© 2026 Botta L et al. *JAMA Network Open*.

	Ewing sarcoma (age <18 y)															
	L				M				X				Overall			
	No. cases	Three-year OS	95%CI		No. cases	Three-year OS	95%CI		No. cases	Three-year OS	95%CI		No. cases	Three-year OS	95%CI	
Central Europe	211	87%	82%	91%	93	71%	61%	79%	3	67%	5%	95%	307	82%	77%	86%
Eastern Europe	69	83%	71%	90%	38	39%	24%	54%	.	.	.	.	107	77%	70%	83%
Northern Europe	29	90%	71%	97%	14	57%	28%	78%	.	.	.	.	43	81%	69%	89%
Southern Europe	130	89%	83%	93%	66	56%	43%	67%	11	100%	.	.	207	77%	71%	81%
UK and Ireland	99	86%	77%	91%	50	36%	23%	49%	12	83%	48%	96%	161	70%	62%	77%
**BENCHISTA Europe**	**538**	**87%**	**84%**	**90%**	**261**	**55%**	**49%**	**61%**	**26**	**88%**	**68%**	**96%**	**825**	**78%**	**75%**	**80%**
Canada	16	100%	.	.	7	43%	10%	73%	1	100%	.	.	24	83%	61%	93%
Japan	4	100%	.	.	1	..	.	.	.	.	.	.	5	80%	20%	97%
Brazil (*)	24	69%	43%	85%	13	25%	6%	50%	3	33%	1%	77%	40	51%	33%	66%

© 2026 Botta L et al. *JAMA Network Open*.

	Rhabdomyosarcoma (age <18 y)																							
	I				II				III				IV				X				Overall			
	**No. cases**	**Three-year OS**	**95%CI**		**No. cases**	**Three-year OS**	**95%CI**		**No. cases**	**Three-year OS**	**95%CI**		**No. cases**	**Three-year OS**	**95%CI**		**No. cases**	**Three-year OS**	**95%CI**		**No. cases**	**Three-year OS**	**95%CI**	
Central Europe	101	95%	89%	98%	44	91%	78%	96%	99	83%	74%	89%	84	40%	30%	51%	26	77%	56%	89%	354	77%	72%	81%
Eastern Europe	39	92%	77%	97%	7	71%	26%	92%	36	69%	52%	82%	39	41%	26%	56%	3	67%	5%	95%	124	72%	65%	78%
Northern Europe	20	100%	.	.	9	67%	28%	88%	15	93%	61%	99%	13	31%	10%	55%	.	.	.	.	57	77%	66%	84%
Southern Europe	74	93%	85%	97%	25	92%	72%	98%	42	74%	58%	85%	47	43%	28%	56%	14	79%	47%	93%	202	78%	72%	83%
UK and Ireland	74	99%	91%	100%	23	87%	65%	96%	65	83%	72%	90%	72	50%	38%	61%	61	79%	66%	87%	295	78%	73%	83%
**BENCHISTA Europe**	**308**	**95%**	**92%**	**97%**	**108**	**87%**	**79%**	**92%**	**257**	**80%**	**75%**	**85%**	**255**	**43%**	**37%**	**49%**	**104**	**78%**	**69%**	**85%**	**1,032**	**77%**	**74%**	**79%**
Canada	15	93%	61%	99%	10	80%	41%	95%	8	75%	31%	93%	25	50%	29%	68%	.	.	.	.	58	70%	57%	80%
Japan (*)	3	100%	.	.	5	75%	13%	96%	6	100%	.	.	6	83%	27%	97%	.	.	.	.	20	89%	63%	97%
Brazil (*)	12	69%	30%	90%	6	50%	11%	80%	12	50%	21%	74%	26	32%	15%	50%	9	14%	1%	46%	65	42%	29%	54%

Footnote:
(*)= potential imprecision due to follow-up issues.
Poland was excluded from Eastern Europe in all cancers except neuroblastoma. Sarcomas are calculated including only cases 0-17 years old (see eTable 1 for country selection). Data on Ewing sarcoma and rhabdomyosarcoma cases from Germany was not made available to the project.
NR=Not reportable for Australian data due to reporting rules to suppress counts<6
°=The M4 category was excluded due to less than 10 cases in the whole BENCHISTA database

© 2026 Botta L et al. *JAMA Network Open*.

eTable 5. Multivariable Model Results for Each Tumor Type

Odds ratio (OR) or Hazard Ratio (HR) with and without stage alongside 95% confidence intervals (CI) and p-values (P)

Neuroblastoma Tier 1 (age 0-14 y)									
**Variable**	**HR**	**P**	**95%CI**		**Variable**	**HR**	**P**	**95%CI**	
**Geographical Area/Country (Ref. Central Europe)**					**Geographical Area/Country (Ref. Central Europe)**				
Australia	0.66	0.224	0.34	1.29	Australia	0.66	0.223	0.34	1.29
Canada	0.61	0.100	0.34	1.10	Canada	0.62	0.111	0.35	1.12
Eastern Europe	1.12	0.386	0.87	1.45	Eastern Europe	1.36	0.021	1.05	1.76
Northern Europe	0.91	0.732	0.54	1.55	Northern Europe	0.81	0.436	0.48	1.37
Southern Europe	0.89	0.398	0.69	1.16	Southern Europe	0.98	0.857	0.75	1.27
UK and Ireland	1.31	0.035	1.02	1.69	UK and Ireland	1.18	0.202	0.91	1.52
					**Stage (Ref: Stage L)**				
					LR	4.45	<0.001	2.32	8.53
					M	18.92	<0.001	10.31	34.70
					MS	11.77	<0.001	5.90	23.47
					Unknown	7.32	<0.001	3.34	16.01

© 2026 Botta L et al. *JAMA Network Open*.

Wilms Tumour (age 0-14 y)									
**Variable**	**HR**	**P**	**95%CI**		**Variable**	**HR**	**P**	**95%CI**	
**Age (Ref. >1-<4yr)**					**Age (Ref. >1-<4yr)**				
<1 year	0.85	0.631	0.45	1.63	<1 year	1.82	0.092	0.91	3.67
5-14 years	1.33	0.202	0.86	2.08	5-14 years	1.07	0.775	0.68	1.67
**Geographical Area/Country (Ref. Central Europe)**					**Geographical Area/Country (Ref. Central Europe)**				
Australia	0.34	0.287	0.05	2.48	Australia	0.34	0.283	0.05	2.46
Canada	1.05	0.936	0.32	3.41	Canada	0.89	0.851	0.27	2.92
Eastern Europe	1.79	0.083	0.93	3.44	Eastern Europe	1.77	0.093	0.91	3.43
Japan	0.98	0.983	0.13	7.15	Japan	0.88	0.897	0.12	6.43
Northern Europe	0.69	0.533	0.21	2.24	Northern Europe	0.62	0.424	0.19	2.02
Southern Europe	1.26	0.421	0.72	2.20	Southern Europe	1.21	0.504	0.69	2.13
UK and Ireland	1.17	0.561	0.69	2.00	UK and Ireland	0.94	0.834	0.55	1.62
					**Stage (Ref: Stage I/y-I)**				
					Stage II/y-II	2.08	0.113	0.84	5.14
					Stage III/y-III	5.62	<0.001	2.60	12.14
					Stage IV	12.87	<0.001	6.22	26.67
					Unknown	6.76	<0.001	2.73	16.71

Medulloblastoma – metastatic (M) stages grouped (age 0-14 y)									
**Variable**	**HR**	**P**	**95%CI**		**Variable**	**HR**	**P**	**95%CI**	
**Sex (Ref. Male)**					**Sex (Ref. Male)**				
Female	0.73	0.015	0.57	0.94	Female	0.78	0.057	0.60	1.01
**Geographical Area/Country (Ref. Central Europe)**					**Geographical Area/Country (Ref. Central Europe)**				
Australia	1.31	0.415	0.68	2.51	Australia	1.36	0.359	0.71	2.60
Canada	1.49	0.178	0.84	2.65	Canada	1.58	0.119	0.89	2.82
Japan	0.62	0.502	0.15	2.51	Japan	0.72	0.643	0.18	2.91
Eastern Europe	1.54	0.034	1.03	2.28	Eastern Europe	1.68	0.011	1.13	2.49
Northern Europe	1.25	0.439	0.71	2.18	Northern Europe	1.32	0.335	0.75	2.31
Southern Europe	1.36	0.054	0.99	1.87	Southern Europe	1.42	0.031	1.03	1.94
UK and Ireland	1.05	0.789	0.74	1.48	UK and Ireland	0.98	0.929	0.69	1.40
					**Stage (Ref. Stage M0)**				
					Stage M1-M4	2.30	<0.001	1.79	2.95
					Unknown	1.69	0.011	1.13	2.53

© 2026 Botta L et al. *JAMA Network Open*.

Osteosarcoma (age < 18 y)									
**Variable**	**OR**	**P**	**95%CI**		**Variable**	**OR**	**P**	**95%CI**	
**Age (Ref. >10yr-<14yr)**					**Age (Ref. >10yr-<14yr)**				
0-4 years	0.96	0.940	0.35	2.64	0-4 years	0.96	0.936	0.34	2.71
5-9 years	0.77	0.213	0.51	1.16	5-9 years	0.77	0.248	0.50	1.20
15-17 years	1.05	0.741	0.78	1.41	15-17 years	1.08	0.625	0.79	1.48
**Geographical Area/Country (Ref. Central Europe)**					**Geographical Area/Country (Ref. Central Europe)**				
Canada	1.62	0.146	0.84	3.13	Canada	1.76	0.134	0.84	3.69
Japan	NE				Japan	NE			
Eastern Europe	1.21	0.453	0.74	1.97	Eastern Europe	1.13	0.664	0.65	1.95
Northern Europe	1.68	0.146	0.84	3.36	Northern Europe	1.51	0.297	0.69	3.30
Southern Europe	0.92	0.656	0.63	1.33	Southern Europe	0.99	0.964	0.66	1.48
UK and Ireland	1.11	0.556	0.78	1.59	UK and Ireland	1.06	0.753	0.73	1.55
					**Stage (Ref. Stage L)**				
					Stage M	3.70	<0.001	2.72	5.03
					Unknown	1.58	0.065	0.97	2.55

Ewing sarcoma (age < 18 y)									
**Variable**	**HR**	**P**	**95%CI**		**Variable**	**HR**	**P**	**95%CI**	
**Age (Ref. >10yr-<14yr)**					**Age (Ref. >10yr-<14yr)**				
0-4 years	0.46	0.020	0.24	0.89	0-4 years	0.61	0.140	0.31	1.18
5-9 years	0.58	0.010	0.38	0.87	5-9 years	0.63	0.034	0.42	0.97
15-17 years	1.36	0.061	0.99	1.86	15-17 years	1.27	0.142	0.92	1.75
**Geographical Area/Country (Ref. Central Europe)**					**Geographical Area/Country (Ref. Central Europe)**				
Canada	0.99	0.988	0.36	2.74	Canada	1.02	0.967	0.37	2.83
Japan	1.33	0.776	0.18	9.68	Japan	1.43	0.722	0.20	10.39
Eastern Europe	2.04	0.001	1.34	3.12	Eastern Europe	1.87	0.004	1.22	2.86
Northern Europe	1.21	0.601	0.60	2.45	Northern Europe	1.09	0.813	0.54	2.21
Southern Europe	1.30	0.200	0.87	1.94	Southern Europe	1.31	0.186	0.88	1.96
UK and Ireland	1.87	0.002	1.27	2.75	UK and Ireland	2.06	<0.001	1.39	3.04
					**Stage (Ref: Stage L)**				
					Stage M	4.26	<0.001	3.16	5.73
					Unknown	0.70	0.555	0.22	2.26

© 2026 Botta L et al. *JAMA Network Open*.

Rhabdomyosarcoma (age < 18 y)									
**Variable**	**HR**	**P**	**95%CI**			**HR**	**P**	**95%CI**	
**Age (Ref. >1yr-<10yr)**					**Age (Ref: >1yr-<10yr)**				
<1 year	1.23	0.444	0.72	2.11	<1 year	1.33	0.294	0.78	2.29
10-14 years	2.13	<0.001	1.60	2.84	10-14 years	1.67	0.001	1.25	2.23
15-17 years	2.17	<0.001	1.55	3.05	15-17 years	1.88	<0.001	1.34	2.66
**Geographical Area/Country (Ref. Central Europe)**					**Geographical Area/Country (Ref. Central Europe)**				
Canada	1.37	0.234	0.81	2.32	Canada	0.92	0.748	0.54	1.56
Eastern Europe	1.53	0.028	1.05	2.23	Eastern Europe	1.34	0.135	0.91	1.96
Northern Europe	1.02	0.939	0.57	1.84	Northern Europe	1.26	0.437	0.70	2.27
Southern Europe	1.04	0.839	0.73	1.48	Southern Europe	1.16	0.418	0.81	1.66
UK and Ireland	0.96	0.793	0.69	1.33	UK and Ireland	0.88	0.463	0.63	1.23
					**Stage (Ref. Stage I)**				
					Stage II	3.49	0.001	1.72	7.10
					Stage III	5.04	<0.001	2.84	8.97
					Stage IV	17.12	<0.001	10.05	29.17
					Unknown	6.13	<0.001	3.17	11.87

Footnote:
HRs are reported for all cancers where the assumption of proportional hazards was confirmed. For Osteosarcoma the assumption failed so ORs calculated using an inverse probability censoring weighting logistic model was used to account for censoring were reported instead. All models were age adjusted and only for medulloblastoma both models were also adjusted by sex. The HRs for medulloblastoma and neuroblastoma are adjusted for age through stratification. Countries excluded: Brazil (all tumours), Japan (neuroblastoma and rhabdomyosarcoma), and Poland (all cancers except neuroblastoma). Sarcomas are calculated including only cases 0-17 years old (see eTable 1 for country selection). Data on Ewing sarcoma and rhabdomyosarcoma cases from Germany was not made available to the project.

© 2026 Botta L et al. *JAMA Network Open*.

eTable 6. Multivariable Age-Stratified Cox Model for Neuroblastoma Using Tier 2 Data and Excluding Germany

Neuroblastoma Tier 2 (age 0-14 y)									
**Variable**	**HR**	**P**	**95%CI**		**Variable**	**HR**	**P**	**95%CI**	
**Geographical Area/Country (Ref. Central Europe)**					**Geographical Area/Country (Ref. Central Europe)**				
Australia	0.65	0.211	0.33	1.28	Australia	0.58	0.122	0.30	1.15
Canada	0.60	0.094	0.33	1.09	Canada	0.60	0.097	0.33	1.10
Eastern Europe	1.11	0.486	0.83	1.47	Eastern Europe	1.33	0.054	1.00	1.79
Northern Europe	0.89	0.684	0.52	1.53	Northern Europe	0.78	0.378	0.45	1.35
Southern Europe	0.88	0.381	0.66	1.17	Southern Europe	0.92	0.598	0.69	1.24
UK and Ireland	1.29	0.072	0.98	1.71	UK and Ireland	1.08	0.608	0.81	1.43
					**Stage (Ref: Stage L)**				
					LR	4.85	<0.001	2.28	10.34
					M	22.30	<0.001	10.98	45.28
					MS	12.53	<0.001	5.60	28.08
					Unknown	14.49	<0.001	6.75	31.14

© 2026 Botta L et al. *JAMA Network Open*.

eReferences

1. Botta L, Didonè F, Lopez-Cortes A, Nieto AC, Desandes E, Hjalgrim LL, et al. International benchmarking of stage at diagnosis for six childhood solid tumours (the BENCHISTA project): a population-based, retrospective cohort study. The Lancet Child & Adolescent Health. 2025;9(2):89-99.

2. Botta L, Gatta G, Didonè F, Lopez Cortes A, Pritchard-Jones K. International benchmarking of childhood cancer survival by stage at diagnosis: The BENCHISTA project protocol. PLoS One. 2022;17(11):e0276997.

3. Aitken JF YD, O'Neill L, Gupta S, Frazier AL. Childhood cancer staging for population registries according to the Toronto Childhood Cancer Stage Guidelines – Version 2. In: Cancer Council Queensland and Cancer Australia: Brisbane A, editor. 2021.

4. Gupta S, Aitken J, Bartels U, Bhakta N, Bucurenci M, Brierley JD, et al. Development of paediatric non-stage prognosticator guidelines for population-based cancer registries and updates to the 2014 Toronto Paediatric Cancer Stage Guidelines. Lancet Oncol. 2020;21(9):e444-e51.

5. Gupta S, Aitken JF, Bartels U, Brierley J, Dolendo M, Friedrich P, et al. Paediatric cancer stage in population-based cancer registries: the Toronto consensus principles and guidelines. Lancet Oncol. 2016;17(4):e163-e72.

© 2026 Botta L et al. *JAMA Network Open*.
