# Supplementary material for: Stage at Diagnosis and International Survival Variation in Childhood Tumors in the BENCHISTA Study
Source: JAMA Netw Open. 2026 Feb 9;9(2):e2556747. doi: 10.1001/jamanetworkopen.2025.56747 (PMC12887745; doi:10.1001/jamanetworkopen.2025.56747)
Supplement: Supplement 2. — Nonauthor Collaborators [file jamanetwopen-e2556747-s002.pdf]

Supplemental Online Content: Nonauthor Collaborators
*First name, last name, and suffix (if applicable) are required and will appear in PubMed.

*Group Name(s): The BENCHISTA Project Working Group

*First Name and Middle Initial(s)	*Last Name	*Suffix (eg, Jr, III)	Academic Degrees	Institution	Location (city, state/province, country)	Role or Contribution, eg, chair, principal investigator	Group (if more than 1 Group listed in the byline) and/or Subgroup (eg, Steering Committee)
Joanne	Aitken			Australian Childhood Cancer Registry	Australia		The BENCHISTA Project Working Group
Leisa	O'Neil			Australian Childhood Cancer Registry	Australia		The BENCHISTA Project Working Group
Danny	Youlden			Australian Childhood Cancer Registry	Australia		The BENCHISTA Project Working Group
Monika	Hackl			Austrian National Cancer Registry	Austria		The BENCHISTA Project Working Group
Ruth	Ladenstein			Children's Cancer Research Institute	Austria		The BENCHISTA Project Working Group
Elizabeth	Van Eycken			Belgian Cancer Registry	Belgium		The BENCHISTA Project Working Group
Nancy	Van Damme			Belgian Cancer Registry	Belgium		The BENCHISTA Project Working Group
Beatriz	De Camargo				Brazil		The BENCHISTA Project Working Group
Marceli	de Oliveira Santos				Brazil		The BENCHISTA Project Working Group
Carlos A.	Lima			RCBP de Aracju	Brazil		The BENCHISTA Project Working Group
Walmiro	Ramos			RCBP de Barretos	Brazil		The BENCHISTA Project Working Group
Lucrecia	Aline Cabral Formigosa			RCBP de Belem	Brazil		The BENCHISTA Project Working Group
Luciana	Ferreira dos Santos			RCBP de Belem	Brazil		The BENCHISTA Project Working Group
Claudina Agnese	Casale			RCBP de Belo Horizonte	Brazil		The BENCHISTA Project Working Group

Supplemental Online Content: Nonauthor Collaborators
*First name, last name, and suffix (if applicable) are required and will appear in PubMed.

***First Name and Middle Initial(s)**	***Last Name**	***Suffix (eg, Jr, III)**	Academic Degrees	Institution	Location (city, state/province, country)	Role or Contribution, eg, chair, principal investigator	Group (if more than 1 Group listed in the byline) and/or Subgroup (eg, Steering Committee)
Gil Patrus	Pena			RCBP de Belo Horizonte	Brazil		The BENCHISTA Project Working Group
Juliana	Nativio			RCBP de Campinas	Brazil		The BENCHISTA Project Working Group
Cyntia Asturian	Laporte			RCBP de Curitiba	Brazil		The BENCHISTA Project Working Group
Cristiana	Santos de Menezes Miranda			RCBP de Distrito Federal	Brazil		The BENCHISTA Project Working Group
Cristiane Bastos	Daniel			RCBP de Distrito Federal	Brazil		The BENCHISTA Project Working Group
Raimunda	Nonata de Paulo			RCBP de Fortaleza	Brazil		The BENCHISTA Project Working Group
Donaldo B.	Veneziano			RCBP de Jahu	Brazil		The BENCHISTA Project Working Group
Angela	Pontes de Aquino			RCBP de Joao Pessoa	Brazil		The BENCHISTA Project Working Group
Paulo Cesar	Fernandes de Souza			RCBP de Mato Grosso	Brazil		The BENCHISTA Project Working Group
Rebeca Valentim	Leite			RCBP de Mato Grosso	Brazil		The BENCHISTA Project Working Group
Zdravka	Valerianova			Bulgarian Cancer Registry	Bulgaria		The BENCHISTA Project Working Group
Dobrin	Konstantinov			Bulgarian Cancer Registry	Bulgaria		The BENCHISTA Project Working Group
Sumit	Gupta			Children Cancer Registry _ POGO	Canada		The BENCHISTA Project Working Group
Jason D.	Pole			Children Cancer Registry _ POGO	Canada		The BENCHISTA Project Working Group
Jan	Stary			Czech National Cancer Registry	Czech Republic		The BENCHISTA Project Working Group

Supplemental Online Content: Nonauthor Collaborators
*First name, last name, and suffix (if applicable) are required and will appear in PubMed.

***First Name and Middle Initial(s)**	***Last Name**	***Suffix (eg, Jr, III)**	Academic Degrees	Institution	Location (city, state/province, country)	Role or Contribution, eg, chair, principal investigator	Group (if more than 1 Group listed in the byline) and/or Subgroup (eg, Steering Committee)
Jaroslav	Sterba			Czech National Cancer Registry	Czech Republic		The BENCHISTA Project Working Group
Lisa L.	Hjalgrim			Danish Childhood Cancer Registry and Department of Paediatric Oncology	Denmark		The BENCHISTA Project Working Group
Jeanette Falck	Winther			Childhood Cancer Research Group, Danish Cancer Institute & Department of Clinical Medicine, Aarhus University and University Hospital	Denmark		The BENCHISTA Project Working Group
Keiu	Paapsi			Estonia National Insitute for Health Development	Estonia		The BENCHISTA Project Working Group
Brigitte	Lacour			French National Registry of Childhood Cancer - Solid Tumours	France		The BENCHISTA Project Working Group
Emmanuel	Desandes			French National Registry of Childhood Cancer - Solid Tumours	France		The BENCHISTA Project Working Group
Jacqueline	Clavel			French National Registry of Childhood Cancer - Hematopoietic Malignancies	France		The BENCHISTA Project Working Group
Claire	Poulalhon			French National Registry of Childhood Cancer - Hematopoietic Malignancies	France		The BENCHISTA Project Working Group
Meike	Ressing			German Childhood Cancer Registry	Germany		The BENCHISTA Project Working Group
Claudia	Truenbach			German Childhood Cancer Registry	Germany		The BENCHISTA Project Working Group
Claudia	Spix			German Childhood Cancer Registry	Germany		The BENCHISTA Project Working Group

Supplemental Online Content: Nonauthor Collaborators
*First name, last name, and suffix (if applicable) are required and will appear in PubMed.

***First Name and Middle Initial(s)**	***Last Name**	***Suffix (eg, Jr, III)**	Academic Degrees	Institution	Location (city, state/province, country)	Role or Contribution, eg, chair, principal investigator	Group (if more than 1 Group listed in the byline) and/or Subgroup (eg, Steering Committee)
Eleni T.	Petridou			Greek Nationwide Registry for Childhood Hematological Malignancies and Solid Tumours (NARECHEM-ST)	Greece		The BENCHISTA Project Working Group
Evdoxia	Bouka			Greek Nationwide Registry for Childhood Hematological Malignancies and Solid Tumours (NARECHEM-ST)	Greece		The BENCHISTA Project Working Group
Zsusanna	Jakab			National Childhood Cancer Registry	Hungary		The BENCHISTA Project Working Group
Miklos	Garami			National Childhood Cancer Registry	Hungary		The BENCHISTA Project Working Group
Rocco	Galasso			Basilicata Cancer Registry	Italy		The BENCHISTA Project Working Group
Giuseppe	Sampietro			Bergamo Cancer Registry	Italy		The BENCHISTA Project Working Group
Patrizia	Piga			Campania Childhood Cancer Registry	Italy		The BENCHISTA Project Working Group
Marcella	Sessa			Campania Childhood Cancer Registry	Italy		The BENCHISTA Project Working Group
Milena M.	Maule			Childhood Cancer Registry of Piedmont	Italy		The BENCHISTA Project Working Group
Carlotta	Sacerdote			Childhood Cancer Registry of Piedmont	Italy		The BENCHISTA Project Working Group
Paola	Ballotari			Cremona & Mantova Cancer Registry	Italy		The BENCHISTA Project Working Group
Luigino	Dal Maso			Friuli Venezia Giulia Cancer Registry, CRO Aviano National Cancer Institute	Italy		The BENCHISTA Project Working Group
Antonina	Torrisi			Integrated Cancer Registry CT-ME-EN	Italy		The BENCHISTA Project Working Group

Supplemental Online Content: Nonauthor Collaborators
*First name, last name, and suffix (if applicable) are required and will appear in PubMed.

***First Name and Middle Initial(s)**	***Last Name**	***Suffix (eg, Jr, III)**	Academic Degrees	Institution	Location (city, state/province, country)	Role or Contribution, eg, chair, principal investigator	Group (if more than 1 Group listed in the byline) and/or Subgroup (eg, Steering Committee)
Rosalia	Ragusa			Integrated Cancer Registry CT-ME-EN	Italy		The BENCHISTA Project Working Group
Luca	Boni			Liguria Cancer Registry, Ospedale Policlinico San Martino IRCCS	Italy		The BENCHISTA Project Working Group
Magda	Rognomi			Monza-Brianza Cancer Registry	Italy		The BENCHISTA Project Working Group
Rosalba	Amodio			Palermo Province Cancer Registry	Italy		The BENCHISTA Project Working Group
Francesco	Cuccaro			Puglia Cancer Registry	Italy		The BENCHISTA Project Working Group
Danila	Bruno			Puglia Cancer Registry	Italy		The BENCHISTA Project Working Group
Antonio G	Russo			Registro Tumori ATS della Citta Metropoltiana di Milano	Italy		The BENCHISTA Project Working Group
Federico	Gervasi			Registro Tumori ATS della Citta Metropoltiana di Milano	Italy		The BENCHISTA Project Working Group
Maria L.	Gambino			Registro Tumori ATS Insubria	Italy		The BENCHISTA Project Working Group
Elisabetta	Borciani			Registro Tumori dell'Emilia-Romagna, Unita di Piacenza	Italy		The BENCHISTA Project Working Group
Maria L.	Michiara			Registro Tumori dell'Emilia-Romagna, Unita di Parma	Italy		The BENCHISTA Project Working Group
Luciana	Mangone			Registro Tumori dell'Emilia-Romagna, Unita di Reggio Emilia	Italy		The BENCHISTA Project Working Group
Gianbattista	Spagnoli			Registro Tumori dell'Emilia-Romagna, Unita di Modena	Italy		The BENCHISTA Project Working Group
Stefano	Ferretti			Registro Tumori dell'Emilia-Romagna, Unita di Ferrara	Italy		The BENCHISTA Project Working Group
Fabio	Falcini			Registro Tumori dell'Emilia-Romagna, Unita della Romagna, IRCCS IRST Meldola	Italy		The BENCHISTA Project Working Group

Supplemental Online Content: Nonauthor Collaborators
*First name, last name, and suffix (if applicable) are required and will appear in PubMed.

***First Name and Middle Initial(s)**	***Last Name**	***Suffix (eg, Jr, III)**	Academic Degrees	Institution	Location (city, state/province, country)	Role or Contribution, eg, chair, principal investigator	Group (if more than 1 Group listed in the byline) and/or Subgroup (eg, Steering Committee)
Eugenia	Spata			Registro Tumori di Ragusa e Caltanissetta	Italy		The BENCHISTA Project Working Group
Sonia	Manasse			Registro Tumori Regione Marche	Italy		The BENCHISTA Project Working Group
Paolo	Coccia			Registro Tumori Regione Marche	Italy		The BENCHISTA Project Working Group
Fabrizio	Stracci			Registro Tumori Umbria	Italy		The BENCHISTA Project Working Group
Daniela	Piras			Registro Tumori Sassari	Italy		The BENCHISTA Project Working Group
Pasquala	Pinna			Registro Tumori Nuoro	Italy		The BENCHISTA Project Working Group
Francesca	Bella			Siracusa Cancer Registry	Italy		The BENCHISTA Project Working Group
Adele	Caldarella			Toscana Cancer Registry	Italy		The BENCHISTA Project Working Group
Teresa	Intrieri			Toscana Cancer Registry	Italy		The BENCHISTA Project Working Group
Tiziana	Scuderi			Registro Tumori Trapani Agrigento	Italy		The BENCHISTA Project Working Group
William	Mantovani			Trento Cancer Registry	Italy		The BENCHISTA Project Working Group
Manuel	Zorzi			Veneto Cancer Registry	Italy		The BENCHISTA Project Working Group
Stefano	Guzzinati			Veneto Cancer Registry	Italy		The BENCHISTA Project Working Group
Deidre	Murray			National Cancer Registry Ireland	Ireland		The BENCHISTA Project Working Group
Tomohiro	Matsuda			National Cancer Centre	Japan		The BENCHISTA Project Working Group

Supplemental Online Content: Nonauthor Collaborators
*First name, last name, and suffix (if applicable) are required and will appear in PubMed.

***First Name and Middle Initial(s)**	***Last Name**	***Suffix (eg, Jr, III)**	Academic Degrees	Institution	Location (city, state/province, country)	Role or Contribution, eg, chair, principal investigator	Group (if more than 1 Group listed in the byline) and/or Subgroup (eg, Steering Committee)
Kayo	Nakata			Osaka Cancer Registry	Japan		The BENCHISTA Project Working Group
Miriam J.	Azzopardi			Malta National Cancer Registry	Malta		The BENCHISTA Project Working Group
Tom Borge	Johannesen			Norwegian Cancer Registry	Norway		The BENCHISTA Project Working Group
Aina H.	Dahlen			Norwegian Cancer Registry	Norway		The BENCHISTA Project Working Group
Bernward	Zeller			Norwegian Cancer Registry	Norway		The BENCHISTA Project Working Group
Jerzy	Kowalczyk			Medical University of Lublin	Poland		The BENCHISTA Project Working Group
Anna	Raciborska			Institute of Mother and Child	Poland		The BENCHISTA Project Working Group
Ana M.	Ferreira			Portuguese Pediatric Cancer Registry	Portugal		The BENCHISTA Project Working Group
Gabriela	Caldas			Portuguese Pediatric Cancer Registry	Portugal		The BENCHISTA Project Working Group
Mihaela	Bucurenci			Romanian Child Cancer Registry	Romania		The BENCHISTA Project Working Group
Daniela	Coza			Romanian Child Cancer Registry	Romania		The BENCHISTA Project Working Group
Vesna	Zadnik			Cancer Registry of Republic of Slovenia	Slovenia		The BENCHISTA Project Working Group
Arantza	Lopez de Munain			Basque Country, Euskadi-CIBERESP Cancer Registry	Spain		The BENCHISTA Project Working Group
Fernando	Alemda-Vich			Childhood and Adolescents Cancer Registry - CISCV	Spain		The BENCHISTA Project Working Group
Noura	Jeghalef-El Karoui			Childhood and Adolescents Cancer Registry - CISCV	Spain		The BENCHISTA Project Working Group

Supplemental Online Content: Nonauthor Collaborators
*First name, last name, and suffix (if applicable) are required and will appear in PubMed.

***First Name and Middle Initial(s)**	***Last Name**	***Suffix (eg, Jr, III)**	Academic Degrees	Institution	Location (city, state/province, country)	Role or Contribution, eg, chair, principal investigator	Group (if more than 1 Group listed in the byline) and/or Subgroup (eg, Steering Committee)
Montse	Puigdemonte			Girona Cancer Registry	Spain		The BENCHISTA Project Working Group
Maria	Jose Sanchez			Granada Cancer Registry	Spain		The BENCHISTA Project Working Group
Nuria	Aragones			Madrid Childhood Cancer Registry	Spain		The BENCHISTA Project Working Group
David	Parra-Blazquez			Madrid Childhood Cancer Registry	Spain		The BENCHISTA Project Working Group
Maria Dolores	Chirlaque			Murcia Cancer Registry	Spain		The BENCHISTA Project Working Group
Marcela	Guevara			Navarra Cancer Registry	Spain		The BENCHISTA Project Working Group
Elena	Pardo			Spanish Registry of Childhood Tumours (RETI-SEHOP), University of Valencia	Spain		The BENCHISTA Project Working Group
Rafael	Peris-Bonet			Spanish Registry of Childhood Tumours (RETI-SEHOP), University of Valencia	Spain		The BENCHISTA Project Working Group
Adela	Canete Nieto			Spanish Registry of Childhood Tumours (RETI-SEHOP), University of Valencia	Spain		The BENCHISTA Project Working Group
Maria	Carulla			Tarragona Cancer Registry	Spain		The BENCHISTA Project Working Group
Päivi	Lähteenmäki			Swedish Childhood Cancer Registry – SCCR, Karolinska Institute	Sweden		The BENCHISTA Project Working Group
Claudia E.	Kuehni			Childhood Cancer Registry, Institute of Social and Preventive Medicine, University of Bern	Switzerland		The BENCHISTA Project Working Group

Supplemental Online Content: Nonauthor Collaborators
*First name, last name, and suffix (if applicable) are required and will appear in PubMed.

***First Name and Middle Initial(s)**	***Last Name**	***Suffix (eg, Jr, III)**	Academic Degrees	Institution	Location (city, state/province, country)	Role or Contribution, eg, chair, principal investigator	Group (if more than 1 Group listed in the byline) and/or Subgroup (eg, Steering Committee)
Shelagh M.	Redmond			Childhood Cancer Registry, Institute of Social and Preventive Medicine, University of Bern	Switzerland		The BENCHISTA Project Working Group
Otto	Visser			The Netherlands Cancer Registry	The Netherlands		The BENCHISTA Project Working Group
Henrike	Karim-Kos			Princess Maxima Center for Pediatric Oncology	The Netherlands		The BENCHISTA Project Working Group
Sarah	Stevens			National Disease Registration Service, Transformation Directorate, NHS England	England		The BENCHISTA Project Working Group
Lucy	Irvine			National Disease Registration Service, Transformation Directorate, NHS England	England		The BENCHISTA Project Working Group
Charles	Stiller			National Disease Registration Service, Transformation Directorate, NHS England	England		The BENCHISTA Project Working Group
Anna	Gavin			Northern Ireland Cancer Registry	Northern Ireland		The BENCHISTA Project Working Group
Deidre	Fitzpatrick			Northern Ireland Cancer Registry	Northern Ireland		The BENCHISTA Project Working Group
Damien	Bennett			Northern Ireland Cancer Registry	Northern Ireland		The BENCHISTA Project Working Group
David S.	Morrison			Scottish Cancer Registry	Scotland		The BENCHISTA Project Working Group
Karen	Smith			Scottish Cancer Registry	Scotland		The BENCHISTA Project Working Group
Dyfed	Wyn Huws			Welsh Cancer Intelligence and Surveillance Unit, Public Health Wales	Wales		The BENCHISTA Project Working Group

Supplemental Online Content: Nonauthor Collaborators
*First name, last name, and suffix (if applicable) are required and will appear in PubMed.

***First Name and Middle Initial(s)**	***Last Name**	***Suffix (eg, Jr, III)**	Academic Degrees	Institution	Location (city, state/province, country)	Role or Contribution, eg, chair, principal investigator	Group (if more than 1 Group listed in the byline) and/or Subgroup (eg, Steering Committee)
Stephanie	Smits			Welsh Cancer Intelligence and Surveillance Unit, Public Health Wales	Wales		The BENCHISTA Project Working Group
Angela	Polanco			NIHR (National Institute for Health and Care Research)	UK		Parent and Patient involvement lead Representative
Giles	Greene			Public Health Wales	Wales		Other Representatives
Riccardo	Capocaccia			Epidemiologia & Prevenzione	Italy		Other Representatives
Andrea	Di Cataldo			Universita degli Studi di Catania	Italy		Other Representatives
Meric	Klein			Belgian Cancer Registry	Belgium		Other Representatives
