# Supplementary material for: Stage at Diagnosis and International Survival Variation in Childhood Tumors in the BENCHISTA Study
Source: JAMA Netw Open. 2026 Feb 9;9(2):e2556747. doi: 10.1001/jamanetworkopen.2025.56747 (PMC12887745; doi:10.1001/jamanetworkopen.2025.56747)
Supplement: Supplement 3. — Data Sharing Statement [file jamanetwopen-e2556747-s003.pdf]

Data Sharing Statement

Botta. Stage at Diagnosis and International Survival Variation in Childhood Tumors in the BENCHISTA Study. *JAMA Netw Open*. Published February 09, 2026. doi:10.1001/jamanetworkopen.2025.56747

Data
Data available: No
